# Transcriptional profiling of Toll-like receptor 2-deficient primary murine brain cells during *Toxoplasma gondii* infection

**DOI:** 10.1371/journal.pone.0187703

**Published:** 2017-11-14

**Authors:** Kousuke Umeda, Sachi Tanaka, Fumiaki Ihara, Junya Yamagishi, Yutaka Suzuki, Yoshifumi Nishikawa

**Affiliations:** 1 National Research Center for Protozoan Diseases, Obihiro University of Agriculture and Veterinary Medicine, Obihiro, Hokkaido, Japan; 2 Division of Animal Science, Department of Agricultural and Life Sciences, Faculty of Agriculture, Shinshu University, Minamiminowa, Nagano, Japan; 3 Research Center for Zoonosis Control, Hokkaido University, Sapporo, Hokkaido, Japan; 4 Graduate School of Frontier Science, The University of Tokyo, Kashiwa, Chiba, Japan; Rutgers University, UNITED STATES

## Abstract

**Background:**

*Toxoplasma gondii* is capable of persisting in the brain, although it is efficiently eliminated by cellular immune responses in most other sites. While Toll-like receptor 2 (TLR2) reportedly plays important roles in protective immunity against the parasite, the relationship between neurological disorders induced by *T*. *gondii* infection and TLR2 function in the brain remains controversial with many unknowns. In this study, primary cultured astrocytes, microglia, neurons, and peritoneal macrophages obtained from wild-type and TLR2-deficient mice were exposed to *T*. *gondii* tachyzoites. To characterize TLR2-dependent functional pathways activated in response to *T*. *gondii* infection, gene expression of different cell types was profiled by RNA sequencing.

**Results:**

During *T*. *gondii* infection, a total of 611, 777, 385, and 1105 genes were upregulated in astrocytes, microglia, neurons, and macrophages, respectively, while 163, 1207, 158, and 1274 genes were downregulated, respectively, in a TLR2-dependent manner. Overrepresented Gene Ontology (GO) terms for TLR2-dependently upregulated genes were associated with immune and stress responses in astrocytes, immune responses and developmental processes in microglia, metabolic processes and immune responses in neurons, and metabolic processes and gene expression in macrophages. Overrepresented GO terms for downregulated genes included ion transport and behavior in astrocytes, cell cycle and cell division in microglia, metabolic processes in neurons, and response to stimulus, signaling and cell motility in macrophages.

**Conclusions:**

To our knowledge, this is the first transcriptomic study of TLR2 function across different cell types during *T*. *gondii* infection. Results of RNA-sequencing demonstrated roles for TLR2 varied by cell type during *T*. *gondii* infection. Our findings facilitate understanding of the detailed relationship between TLR2 and *T*. *gondii* infection, and elucidate mechanisms underlying neurological changes during infection.

## Introduction

*Toxoplasma gondii* is an obligate intracellular parasite of warm-blooded animals. It is generally assumed that approximately 30% of the world's human population is infected by this parasite [1]. While infection with *T*. *gondii* causes no symptoms in healthy adult humans, it causes severe symptoms such as toxoplasmic encephalitis in immunocompromised patients, including individuals diagnosed with AIDS [2]. Moreover, if a woman receives her first exposure to *T*. *gondii* while pregnant, the fetus can be congenitally infected. Congenital toxoplasmosis is associated with fetal death and abortion, and can cause chorioretinitis, hydrocephalus, or intracranial calcifications [3].

In its intermediate host, *T*. *gondii* proliferates as two different asexual stages, termed tachyzoite and bradyzoite. Tachyzoite is a stage of rapid growth occurring during initial acute infection [4]. The majority of tachyzoites are efficiently eliminated by interferon-gamma (IFN-γ)-dependent cell-mediated immune responses. However, in some sites, including the central nervous system (CNS), tachyzoites differentiate into bradyzoites that eventually establish a chronic infection [5]. Bradyzoite is a prolonged slow-growing cyst stage that persists for the lifetime of the host [6]. This chronic infection causes neurologic and behavioral abnormalities secondary to inflammation and loss of brain parenchyma [7].

*T*. *gondii* is capable of infecting any nucleated cell *in vitro*, including astrocytes, microglia, and neurons [8]. However, suggested roles for these brain cells during *T*. *gondii* infection are quite different. Previous *in vivo* studies showed that parasitic cysts in the brain are found almost exclusively within neurons, suggesting neurons are the primary target cell for *T*. *gondii* [9, 10]. Astrocytes play contradictory roles as either parasite proliferation recipients or protective immune response activators, with each role likely depending on the degree of infection [11]. Microglia are often considered to be the tissue-resident macrophages of the brain [12]. Upon infection with *T*. *gondii*, microglia exhibit hypermotility similar to that described for dendritic cells, suggesting microglia may also act as “Trojan horses” to facilitate dissemination of the parasite [13, 14].

Toll-like receptor 2 (TLR2), an important pattern recognition receptor of pathogen-associated molecular patterns, plays a critical role in mammalian innate immune responses [15, 16]. However, the importance of TLR2 in resistance against *T*. *gondii* remains controversial. Mun et al. [17] reported that TLR2-deficient mice failed to survive against *T*. *gondii* infection, and concluded that TLR2 is an essential molecule for protective immunity against *T*. *gondii*. In contrast, Debierre-Grockiego et al. [18] reported no effect of single TLR2-knockout on the survival of mice during infection, and went on to describe varying roles for TLRs during *T*. *gondii* infection depending on the genetic background of mice, infective inoculums, and parasite strain used. TLR2 expression has been observed in astrocytes, microglia, and neurons, where it may play roles in the development and regulation of CNS inflammation, neurodegeneration, and trauma [19]. However, much remains unknown about the relationship between *T*. *gondii* infection-induced neurological disorders and the function of TLR2 in the brain.

To characterize functions of the TLR2 signaling pathway in different CNS cell types, we obtained primary cultured astrocytes, microglia, neurons, and peritoneal macrophages from wild-type and TLR2-deficient mice. These cells were exposed to *T*. *gondii* tachyzoites, and their gene expression was profiled by RNA-sequencing (RNA-seq). Our results showed that during *T*. *gondii* infection, cells differentially expressed many genes associated with immune responses, cell activation, and cell metabolism in a TLR2-dependent manner. To our knowledge, this is the first transcriptomic study focusing on TLR2 in different CNS cell types infected with the parasite. Our findings provide basic information on the relationship between the TLR2 pathway and *T*. *gondii*, and facilitate better understanding of mechanisms underlying neurological changes occurring during *T*. *gondii* infection.

## Materials and methods

### Ethics statement

This study was performed in strict accordance with recommendations of the Guide for the Care and Use of Laboratory Animals of the Ministry of Education, Culture, Sports, Science and Technology, Japan. The protocol was approved by the Committee on the Ethics of Animal Experiments at Obihiro University of Agriculture and Veterinary Medicine (permit numbers 23–56, 24–10, 25–62, and 26–67). All surgeries were performed under isoflurane anesthesia with every effort made to minimize animal suffering.

### Animals

C57BL/6J mice, 6–8 weeks of age, were obtained from Clea Japan (Tokyo, Japan). Homozygous *TLR2*-knockout (*Tlr2*^-/-^) mice were a kind gift from Dr. Satoshi Uematsu and Dr. Shizuo Akira (Osaka University, Osaka, Japan) [15]. To obtain primary brain cells, 8 fetal mice were harvested from one adult female of each genotype 17–18 days after mating. Fetal mice were used without distinction of sex. Peritoneal macrophages were collected from one adult female mouse of each genotype. All animals were housed under specific-pathogen-free conditions in the animal facility of the National Research Center for Protozoan Diseases at Obihiro University of Agriculture and Veterinary Medicine, Hokkaido, Japan.

### Preparation of *T*. *gondii* tachyzoites

Tachyzoites of *T*. *gondii* (PLK strain, type II) were maintained by serial passage on monolayers of Vero cells at 37°C in humidified air with 5% CO_2_. Parasites and host cell debris were washed by centrifugation and resuspended in cold phosphate-buffered saline (PBS). Clustered cells and debris were removed by repeatedly passing through a 27-gauge needle and filtering with a 5.0-μm pore-size filter (Millipore, MA, USA).

### Astrocyte cultures

Astrocytes were obtained from brain cortices of fetal mice (age, E17–18) according to a previously described procedure [20], with some modifications. Fetal mice were decapitated and brains were removed. After removing meninges, cortices were mechanically dissociated into a single-cell suspension in Dulbecco's Modified Eagle’s Medium (DMEM; Sigma-Aldrich, Tokyo, Japan) containing 0.25% trypsin and 0.01% DNase. After incubation at 37°C for 10 min, dissociated cells were washed and suspended in DMEM/F-12 (Gibco-BRL, CA, USA) supplemented with penicillin-streptomycin (100 U/ml of penicillin and 100 μg/mL of streptomycin; Sigma-Aldrich), 10% fetal bovine serum (FBS; Columbia Biosciences, MD, USA), and G-5 Supplement (Gibco-BRL). Cells were plated in 75-cm^2^ flasks at a density of 2 × 10^6^ cells/flask and incubated at 37°C in a humidified 5% CO_2_ and 95% air atmosphere. Culture medium was changed every 3 days until cultures reached confluence, usually after 7–8 days. Astrocyte monolayers were washed and dissociated with 0.25% trypsin–EDTA solution. Dissociated astrocytes were centrifuged at 4°C and 500 × *g* for 5 min, washed in DMEM/F-12 supplemented with 10% FBS and G-5 Supplement, and reseeded in 24-well plates at a density of 2 × 10^5^ cells/well. Primary astrocytes were allowed to grow for 16 h before infection. Approximately 95% of cultured cells were identified as astrocytes based on positive staining for glial fibrillary acidic protein (S1 Fig).

### Microglia cultures

Microglia were obtained using a procedure similar to that used for astrocytes, with some modifications. Dissociated brain cells were washed and suspended in DMEM/F-12 supplemented with penicillin-streptomycin, 10% FBS, and 10 ng/ml of granulocyte–macrophage colony-stimulating factor (R&D Systems, MN, USA). Cells were plated in 75-cm^2^ flasks at a density of 4 × 10^6^ cells/flask and culture medium was changed every 3 days. After 10–11 days of incubation, microglia were detached from the astrocyte monolayer by pipetting. Suspended cells were centrifuged and reseeded in 24-well plates at a density of 2 × 10^5^ cells/well. Primary microglia were allowed to grow for 16 h before infection. Approximately 95% of cultured cells were identified as microglia based on positive staining for CD11b (BD Pharmingen, CA, USA; S2 Fig).

### Neuron cultures

Neurons were obtained according to a previously described procedure [21], with some modifications. Brain cells were suspended in DMEM/F-12 supplemented with penicillin-streptomycin and B27 supplement, and then plated in 12-well plates at a density of 1 × 10^6^ cells/well. Culture medium was changed every 3 days. Primary neurons were allowed to grow for 8 days before infection. To compare proportion of neurons in cultured cells, mean normalized counts for a neuronal marker *Rbfox3*, also called *NeuN*, in RNA-seq data were compared between uninfected wild-type and uninfected *Tlr2*^-/-^ samples, and there was no significant difference with a false discovery rate (FDR) of 0.12.

### Peritoneal macrophage cultures

Peritoneal macrophages were isolated from peritoneal cavities 4 days after injection of 1 mL of 4.05% thioglycollate medium. Peritoneal exudate cells were harvested by lavage with 5 ml of ice-cold PBS and filtered through a 40-μm cell strainer to remove cell aggregates and debris. After centrifugation at 1000 × *g* for 5 min, pelleted cells were resuspended in DMEM supplemented with 10% FBS and penicillin-streptomycin, and seeded in a 96-well plate at a density of 4 × 10^5^ cells/well. Macrophages were allowed to grow for 16 h before infection. Approximately 90% of cultured cells were identified as macrophages based on positive staining for CD11b (S3 Fig).

### In vitro infection and RNA extraction

Primary cultured cells were infected with purified *T*. *gondii* tachyzoites. Multiplicities of infection were 1, 1, 0.2, and 0.25 for astrocytes, microglia, neurons, and macrophages, respectively. After 24 h of infection, total RNA was extracted with TRI reagent (Sigma-Aldrich) according to the manufacturer’s instruction. The experiment was performed in triplicate wells.

### RNA-seq analysis

Transcriptome sequencing was performed as described in our previous study [22]. Briefly, 1 μg of total RNA was subjected to poly-A selection. Sequencing libraries were constructed with a TruSeq RNA Sample Prep Kit (Illumina, CA, USA), while 36-bp single-end sequencing was performed with the Illumina Genome Analyzer IIx and TruSeq SBS Kit v5-GA (36-cycle) (Illumina) according to the manufacturer’s instructions. All treatments and subsequent analyses were performed for individual transcripts.

Sequence tags were aligned using TopHat (version 1.3.3 doi:10.1093/bioinformatics/btp120) and general transfer format (gtf) data (Mus_musculus.GRCm38.69), as previously described [22]. Raw sequence reads were mapped to the mouse genome (mm10) with an allowance of two mismatches. The reads were also mapped to the *T*. *gondii* genome (ToxoDB-34_TgondiiME49) using general feature format (gff) data obtained from ToxoDB [23].

### Identification of differentially expressed genes (DEGs)

Based on mapping results, normalized transcription profiles were estimated using the DESeq package in R software [24]. Mean normalized counts were calculated from raw read counts for each transcript. MGI ID and gene ontology (GO) data were obtained from the Mouse Genome Informatics database [25], and then integrated into the estimated expression profiles, together with gene biotypes extracted from gtf data. The expression of each gene was compared between infected and uninfected cells using DESeq. DEGs were identified as genes with a two-fold change (log2 fold-change > 1 or < -1) and < 0.05 FDR.

### Identification of TLR2-dependent genes

DEGs were compared between wild-type and *Tlr2*^-/-^ cells to identify which genes were differentially expressed in a TLR2-dependent manner. DEGs upregulated or downregulated in wild-type but not *Tlr2*^-/-^ animals were regarded as TLR2-dependent genes. Such genes were functionally categorized by GO term enrichment analysis. Statistical overrepresentation of GO terms for selected genes were compared with reference genes (all genes; 37315 genes) using the GOseq package in R software [26]. Functional annotation charts of enriched GO terms were generated using GO terms associated with biological process. Only GO terms with a < 0.05 p-value were used to represent functional enrichment. Furthermore, TLR2-dependent genes were compared among brain cells (i.e. astrocytes, microglia, and neurons) and between phagocytic cells (i.e. microglia and macrophages). TLR2-dependent genes in each cell type were also subjected to GO analysis as described above.

To exclude minor expressed genes from consideration, highly upregulated or downregulated TLR2-dependent DEGs were defined by cut-off values of > 100 mean normalized counts in infected wild-type for upregulated genes, and > 50 mean normalized counts in uninfected wild-type for downregulated genes. When gene expression levels were compared among all four groups (i.e. uninfected wild-type, infected wild-type, uninfected *Tlr2*^-/-^, and infected *Tlr2*^-/-^), counts for uninfected *Tlr2*^-/-^ and infected *Tlr2*^-/-^ were normalized by multiplying counts in DEseq comparison between “uninfected *Tlr2*^-/-^ and infected *Tlr2*^-/-^” by fold-changes between “uninfected wild-type vs uninfected *Tlr2*^-/-^”.

### Cytokine and PGE_2_ analyses

Astrocytes, microglia and peritoneal macrophages were infected with *T*. *gondii* tachyzoites as described above. For astrocytes, culture supernatants at 48 h after infection were collected to measure production of interleukin (IL)-6. For microglia, concentration of IL-12p40, IL-6 and IL-10 at 24 h after infection and concentration of IL-1β at 48 h after infection were measured. For macrophages, IL-6 and IL-12p40 levels were measured at 24 h after infection. Concentration of these cytokines were determined using a set of ELISA kits (BD Pharmingen), according to the manufacturer’s recommendations. Supernatants of astrocytes were also tested for Prostaglandin E2 (PGE_2_) with an enzyme immunoassay kit (Cayman Chemical Co., MI, USA).

### Nitric oxide (NO) and cytokine analyses after stimulation by IFN-γ

Microglia from wild-type and *Tlr2*^-/-^ mice were infected with *T*. *gondii* tachyzoites as described above and then stimulated with recombinant IFN-γ (10 ng/mL). Culture supernatants were harvested at 24 h after infection to determine concentration of NO using a nitrite/nitrate assay kit (Cayman Chemical Co.), according to the manufacturer’s recommendations. Concentration of IL-12p40 in the supernatants was also determined as described above.

### Statistical analysis

For the resulting top 30 GO terms in each analysis, the number of DEGs associated with a GO term was compared between wild-type and *Tlr2*^-/-^. Statistically significant differences were determined by Fisher’s exact test (*p* < 0.05). For analyses of production of cytokines, PGE_2_ and NO, Student’s t-test was performed to determine significant differences between the two genotypes (*p* < 0.05).

## Results and discussion

### TLR2-dependent gene expression induced by *T*. *gondii* in astrocytes

**Fig 1:**
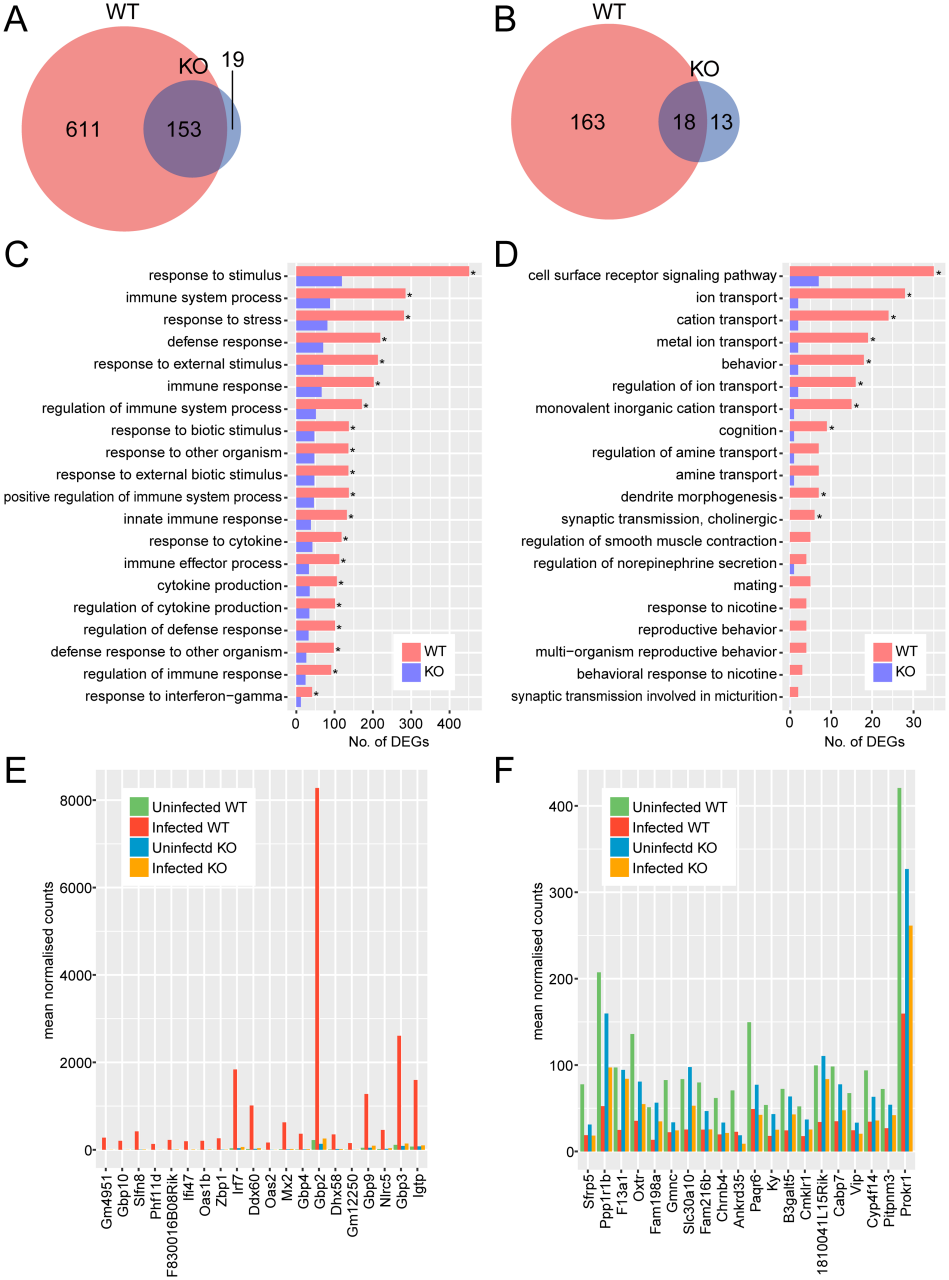
Comparison of transcriptional profiles for *Tlr2*^-/-^ and wild-type astrocytes during *T*. *gondii* infection. Upregulated (A, C, E) and downregulated (B, D, F) genes were identified as genes with 2-fold change and < 0.05 FDR in DESeq analysis comparing infected and uninfected cells. (A, B) Venn diagrams were created to compare DEGs with increased and decreased abundance between *Tlr2*^-/-^ and wild-type. (C, D) To explore the function of DEGs analyzed in the Venn diagram, GO term enrichment analysis was performed. Asterisks represent significant differences with *p* < 0.05 in Fisher’s exact test. (E, F) Expression of top 20 highly upregulated or downregulated TLR2-dependent genes. TLR2-dependent DEGs were ranked according to fold-changes between infected and uninfected wild-type. WT, wild-type; KO, *Tlr2*^-/-^.

RNA-seq was performed to profile the gene expression of primary cultured astrocytes during *T*. *gondii* infection. Comparing DEGs between infected and uninfected wild-type astrocytes, 764 genes were more abundant and 181 were less abundant in infected cells (Fig 1A and 1B). Compared with wild-type cells, the number of DEGs was significantly decreased in *Tlr2*^-/-^ astrocytes, as 172 genes were more abundant and 31 less abundant in infected cells compared with uninfected. DEGs were considered to be TLR2-dependent if they were differentially expressed in wild-type but not *Tlr2*^-/-^ cells. In total, 611 upregulated and 163 downregulated genes were TLR2-dependent in astrocytes (Fig 1A and 1B).

To overview their function, TLR2-dependent DEGs were subjected to GO enrichment analysis. Among upregulated TLR2-dependent genes, overrepresented GO terms were primarily related to stress and immune responses, and cytokines (Fig 1C). Proposed functions of downregulated genes were associated with cell surface receptor signaling pathways, ion transport, and behavior (Fig 1D). These results suggest that TLR2 is important for promoting cytokine-mediated immune responses against *T*. *gondii* in astrocytes, and may also be related to behavioral disorders observed within individual animals during *T*. *gondii* infection.

To analyze pathways regulated by TLR2 in more detail, TLR2-dependent genes were ranked according to fold-changes between infected and uninfected wild-type, and expression of the top 20 genes was compared (Fig 1E and 1F; S1 and S2 Tables). The top 20 upregulated genes included many IFN-inducible genes (such as *F830016B08Rik*, *Gbp2*, *Gbp3*, *Gbp4*, *Gbp9*, *Gbp10*, *Gm4951*, *Gm12250*, *Ifi47*, *Igtp*, *Mx2*, and *Slfn8*). Among IFNs, only IFN-β (encoded by *Ifnb1*) was significantly upregulated in wild-type but not *Tlr2*^-/-^ although the expression level was very low (S3 Table). Perhaps expression of these IFN-inducible genes is induced by INF-β in a TLR2-dependent signaling pathway. Regardless, p65 guanylate-binding proteins (GBPs), including GBP2 and GBP3, localize at the parasitophorous vacuole of *T*. *gondii* where they directly contribute to control of the parasite [27,28]. GBPs, including GBP4, GBP9, and GBP10, were reported to be more abundant in acute, but not chronic infection [29]. Subsequent investigation of gene-deficient mouse strains showed that p47 GTPases, such as IRG-47 (encoded by *Ifi47*) and IGTP, are essential during infection with intracellular pathogens such as *Listeria monocytogenes*, *Mycobacterium tuberculosis*, or *T*. *gondii* in mice [30,31]. The IFN-inducible GTPase family (*Gbp4*, *Gbp8*, *Iigp1*, *Igtp*, and *Tgtp2*) has also been reported to be significantly upregulated in the brains of mice infected with *T*. *gondii* [22]. Our present results are consistent with these previous studies, suggesting upregulation of these genes was dependent on TLR2. In addition, upregulated genes included those related to antiviral activity in both a positive (*Mx2*, *Oas1b*, *Oas2*, *Slfn8*, *Zbp1*) and negative (*Nlrc5*) manner. For example, 2'-5'-oligoadenylate synthases (OASs) mediate RNA degradation as part of the innate antiviral immunity pathway [32]. Thus, differential expression of these genes may suggest immune responses against viral infection were also affected by *T*. *gondii* infection in a TLR2-dependent manner. An early study reported that a virulent strain of *T*. *gondii* (RH) induced high levels of antiviral activity in the serum and peritoneal fluid of mice, and that prior inoculation with avirulent *T*. *gondii* (ME49) induced *in vivo* antiviral protection against a neurotropic virus, Mengo virus [33]. Few genes were remarkably downregulated in a TLR2-dependent manner during *T*. *gondii* infection, partially because their expression was affected just by the deficiency of TLR2. Of the genes downregulated, *F13a1* (encoding coagulation factor XIII A chain) was downregulated only in infected wild-type astrocytes. Notably, coagulation factor XIII normally acts to stabilize fibrin clots by cross-linking fibrin monomers [34], with low levels of this enzyme being correlated with clinical severity of human *Plasmodium falciparum* malaria [35]. In addition, coagulation factor XIII A is produced by astrocytes and microglia in goldfish, where it has been implicated in the regeneration of retina and optic nerve [36]. Hence, downregulation of this molecule is perhaps related to decreased neuronal function during toxoplasmosis.

### TLR2-dependent gene expression induced by *T*. *gondii* in microglia

**Fig 2:**
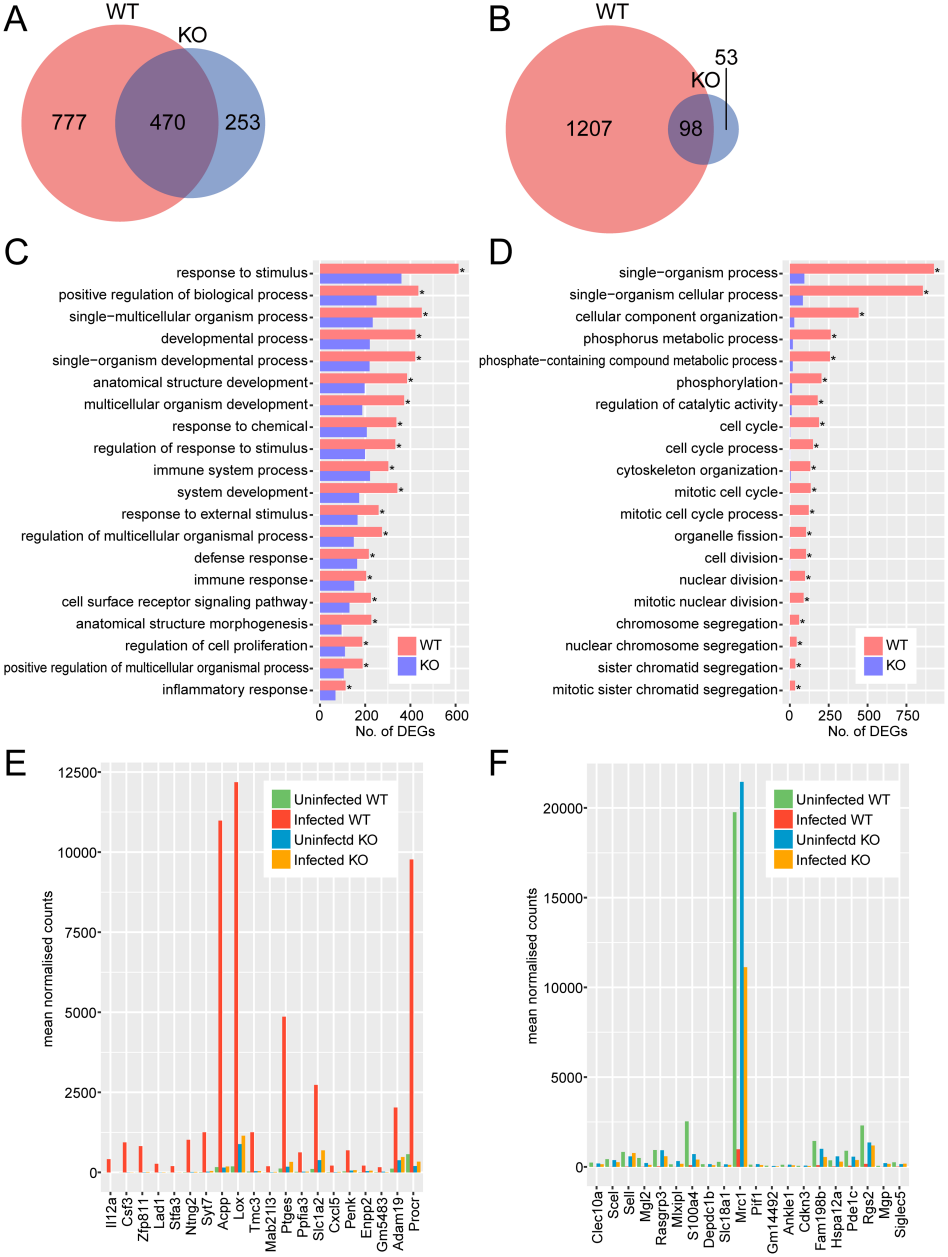
Comparison of transcriptional profiles of *Tlr2*^-/-^ and wild-type microglia during *T*. *gondii* infection. Upregulated (A, C, E) and downregulated (B, D, F) genes were identified as genes with 2-fold change and < 0.05 FDR in DESeq analysis comparing infected and uninfected cells. (A, B) Venn diagrams comparing DEGs with increased and decreased abundance between *Tlr2*^-/-^ and wild-type mice. (C, D) To explore the function of DEGs analyzed in the Venn diagram, GO term enrichment analysis was performed. Asterisks represent significant differences with *p* < 0.05 in Fisher’s exact test. (E, F) Expression of top 20 genes highly upregulated or downregulated in a TLR2-dependent manner. TLR2-dependent DEGs were ranked according to fold-changes between infected and uninfected wild-type. WT, wild-type; KO, *Tlr2*^-/-^.

The number of DEGs in *Tlr2*^-/-^ microglia was much lower than in wild-type. Wild-type microglia upregulated 1247 genes and downregulated 1305 genes during *T*. *gondii* infection, while *Tlr2*^-/-^ microglia upregulated 723 genes and downregulated 151 genes (Fig 2A and 2B). Of these DEGs, 777 upregulated and 1207 downregulated genes were expressed in a TLR2-dependent manner (Fig 2A and 2B).

Upregulated TLR2-dependent DEGs were associated with stress and immune responses, as well as developmental processes (Fig 2C). For downregulated DEGs, GO terms associated with phosphorylation and cell cycle were overrepresented (Fig 2D). Stress and immune responses were also overrepresented in astrocytes, while other overrepresented GO terms were different to those observed in astrocytes. Likely, this is because both astrocytes and microglia play major roles in the immune response, but their detailed functions are quite different. Astrocytes act as neuroprotective barriers to inflammatory cells and infectious agents, and restrict the spread of invading microbial agents such as *T*. *gondii* into the CNS parenchyma. Whereas, microglia are motile and phagocytic cells that function as macrophages within the CNS [37–39]. Microglial proliferation is a major component in the evolution of chronic neurodegeneration [40]. The regulation of microglia proliferation during *T*. *gondii* infection may also be associated with neurological disorders observed in the brain.

Microglia are important for the production of inflammatory cytokines within the brain, as well as serving as antigen-presenting cells similar to dendritic cells and macrophages. Genes encoding cytokines/chemokines (*Csf3*, *Cxcl5*, *Il12a*) were among the highly upregulated TLR2-dependent DEGs (Fig 2E, S4 Table). Notably, TLR2 is required for the production of C-X-C motif chemokine 5 (CXCL5) in the brain in response to *Staphylococcus aureus* [41], and for the production of IL-12 by microglia in response to herpes simplex virus [42]. Activation of TLR2 by bacterial lipoprotein upregulates the production of granulocyte-colony stimulating factor (G-CSF, encoded by *Csf3*) by neutrophils [43]. Consistent with these studies, our results suggested that TLR2 was largely responsible for the production of these proteins in microglia. In addition, some TLR2-dependent upregulated DEGs (*Adam19*, *Mab21l3*, *Ntng2*, *Penk*, *Slc1a2*, *Syt7*) were associated with neural development and synaptic function [44–49], suggesting that microglia affected CNS neural functions in a TLR2-dependent manner during *T*. *gondii* infection. In a recent study of inflammatory mechanisms and neural circuit function, TLR2 was reported to have an important role in the development of sickness behaviors via stimulation of hypothalamic microglia to promote neuronal activation [50]. In contrast, some genes encoding C-type lectins (*Clec10a*, *Mgl2*, *Mrc1*, *Sell*) were clearly downregulated in infected wild-type microglia but not in *Tlr2*^-/-^ microglia (Fig 2F, S5 Table). Both TLRs and C-type lectin receptors are pattern recognition receptors, thus these results may suggest that the recognition pathway dependent on C-type lectins is suppressed after pathogen recognition by TLR2.

### TLR2-dependent gene expression induced by *T*. *gondii* in neurons

**Fig 3:**
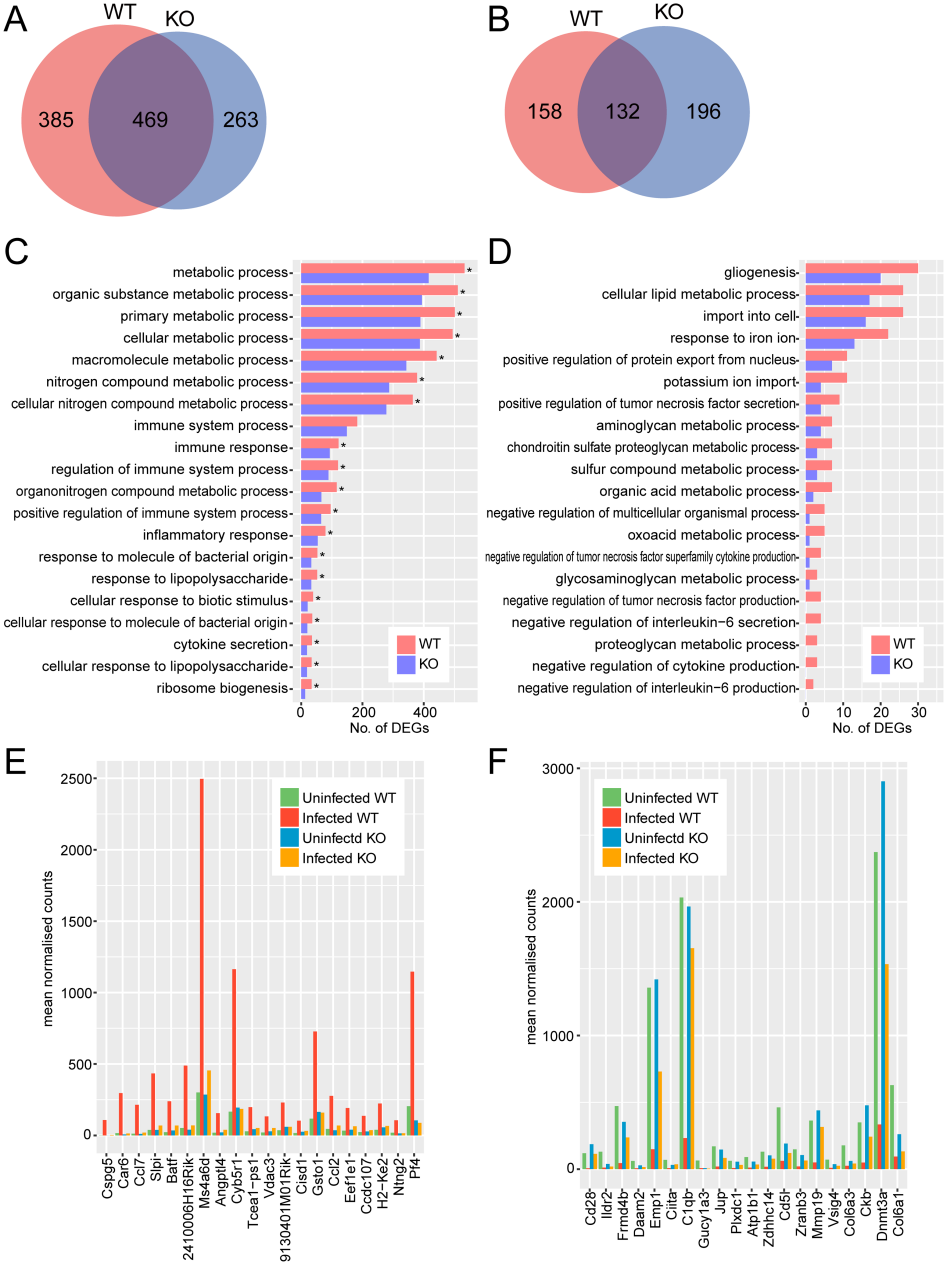
Comparison of transcriptional profiles of *Tlr2*^-/-^ and wild-type neurons during *T*. *gondii* infection. Upregulated (A, C, E) and downregulated (B, D, F) genes were identified as genes with 2-fold change and < 0.05 FDR in DESeq analysis comparing infected and uninfected cells. (A, B) Venn diagrams comparing DEGs with increased and decreased abundance between *Tlr2*^-/-^ and wild-type neurons. (C, D) To explore the function of DEGs analyzed in the Venn diagram, GO term enrichment analysis was performed. Asterisks represent significant differences with *p* < 0.05 in Fisher’s exact test. (E, F) Expression of top 20 genes highly upregulated or downregulated in a TLR2-dependent manner. TLR2-dependent DEGs were ranked according to fold-changes between infected and uninfected wild-type. WT, wild-type; KO, *Tlr2*^-/-^.

Wild-type neurons had 854 upregulated and 290 downregulated genes, while *Tlr2*^-/-^ neurons had 732 upregulated and 328 downregulated genes (Fig 3A and 3B). Notably, the difference in the number of DEGs between wild-type and *Tlr2*^-/-^ was relatively small compared to other cell types examined. A total of 358 genes were upregulated and 158 genes were downregulated in a TLR2-dependent manner (Fig 3A and 3B).

Overrepresented GO terms for upregulated DEGs were primarily associated with stress and immune responses, and metabolic processes (Fig 3C). For TLR2-dependently downregulated DEGs, overrepresented GO terms were associated with metabolic processes and negative regulation of cytokine production, however numbers of DEGs for each GO term were not significantly different between wild-type and *Tlr2*^-/-^ (Fig 3D).

Expression profiles were also compared for each gene. *Serpinb2* (encoding serine protease inhibitor b2) was highly upregulated in a TLR2-dependent manner (Fig 3E, S6 Table). Previous studies have shown inhibited activity of *T*. *gondii* serine protease by some serine protease inhibitors, resulting in the restricted invasion and replication of the parasite and decreased parasite viability [51–53]. Notably, genes associated with inflammatory response (*Ptges*, *Lcn2*, *Nlrp3*, *Chi3l1*) were highly upregulated only in wild-type neurons. Upregulation of *Ptges*, which encodes prostaglandin E synthase, is notable considering the results of a previous study suggesting that *T*. *gondii* induces PGE_2_ in macrophages [54]. In addition, *Hpgd* expression was highly downregulated (Fig 3F, S7 Table). Hydroxyprostaglandin dehydrogenase 15-(NAD), which is encoded by *Hpgd*, is involved in prostaglandin inactivation [55]. Considering these factors together, the parasite appears to induce secretion of the immunosuppressive molecule PGE_2_ to enable its survival within the host. NLRP3, the sensor component of the NLRP3 inflammasome, plays a crucial role in innate immunity and inflammation. *T*. *gondii* activates both NLRP1 and NLRP3 inflammasomes *in vivo*, and the activation of these sensors has been implicated in host resistance to toxoplasmosis [56]. Lipocalin 2 (encoded by *Lcn2*) is involved in innate immunity, possibly by sequestrating iron to limit the growth of bacteria and *Plasmodium* parasites [57,58], but its function during *T*. *gondii* infection is unknown. Interestingly, some downregulated genes were associated with growth factor signaling (*Reps2*, *Eps8*, *Igf1*) [59,60] or axon guidance and neuronal migration (*Plxnc1*, *Plxnb3*) [61,62] (Fig 3F). Differential expression of these genes perhaps indicates that TLR2 signaling is needed to maintain normal neuronal function during infection. Although TLR2 has been reported to be upregulated in neurons in response to glucose deprivation or IFN-γ stimulation [63], so far, the relationship between this pathway and *T*. *gondii* infection has not been revealed.

### TLR2-dependent gene expression induced by *T*. *gondii* in macrophages

**Fig 4:**
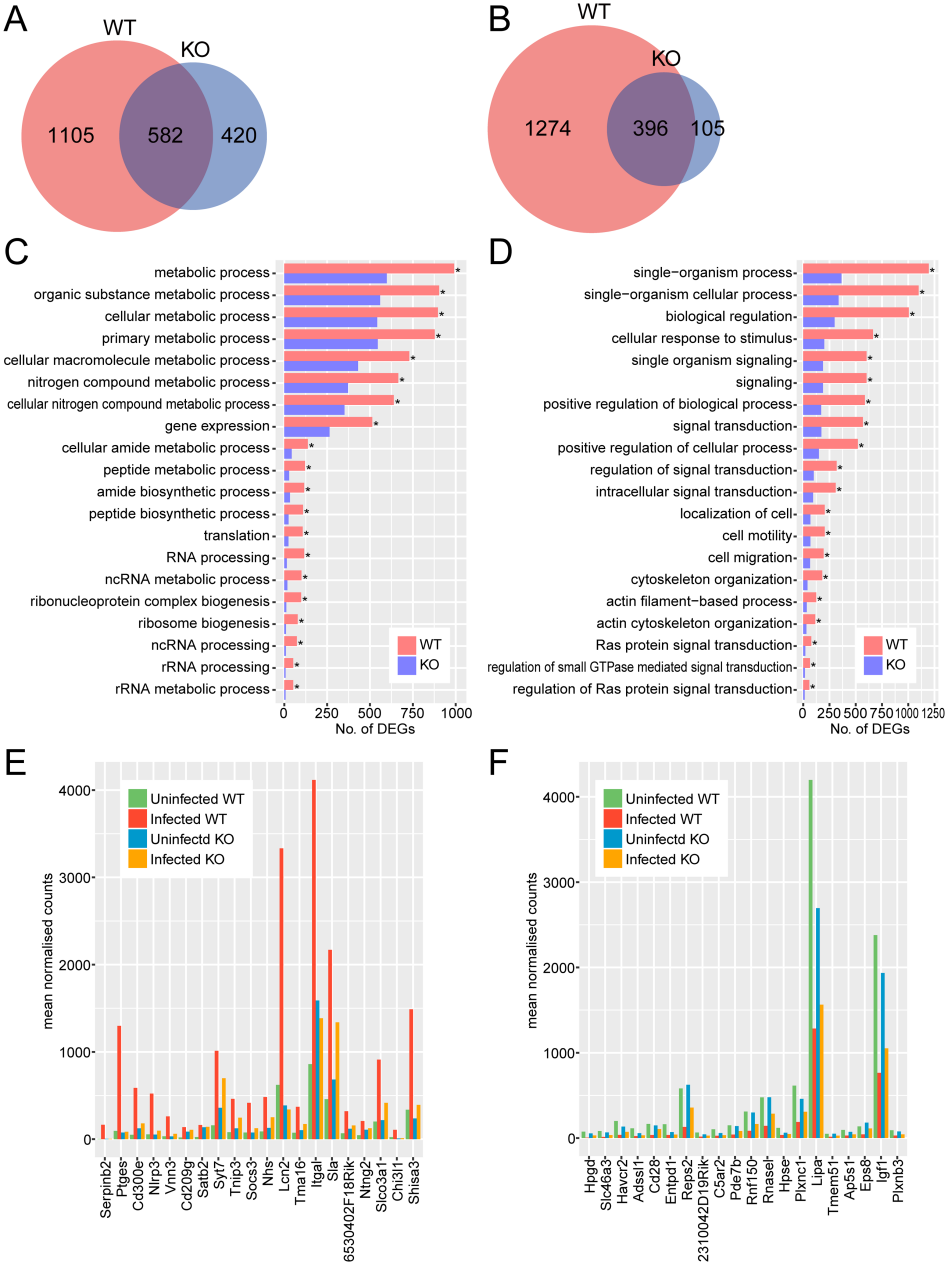
Comparison of transcriptional profiles of *Tlr2*^-/-^ and wild-type macrophages during *T*. *gondii* infection. Upregulated (A, C, E) and downregulated (B, D, F) genes were identified as genes with 2-fold change and < 0.05 FDR in DESeq analysis comparing infected and uninfected cells. (A, B) Venn diagrams comparing DEGs with increased and decreased abundance between *Tlr2*^-/-^ and wild-type macrophages. (C, D) To explore the function of DEGs analyzed in the Venn diagram, GO term enrichment analysis was performed. Asterisks represent significant differences with *p* < 0.05 in Fisher’s exact test. (E, F) Expression of top 20 highly upregulated or downregulated TLR2-depdent genes. TLR2-dependent DEGs were ranked according to fold-changes between infected and uninfected wild-type. WT, wild-type; KO, *Tlr2*^-/-^.

Comparing gene expression profiles between infected and uninfected wild-type macrophages, 1687 genes were more abundant and 1670 were less abundant in infected cells. In infected *Tlr2*^-/-^ macrophages, 1002 genes were more abundant and 501 were less abundant (Fig 4A and 4B). Macrophages expressed 1105 upregulated and 1274 downregulated genes in a TLR2-dependent manner (Fig 4A and 4B).

Interestingly, for upregulated TLR2-dependent genes in macrophages, GO terms associated with stress and immune responses did not rank in the top 20, unlike other cell types examined (Fig 4C). Instead, the top 20 terms were mainly associated with metabolic processes and gene expression. However, all of the top 20 GO terms for genes upregulated in a TLR2-independent manner (upregulated genes commonly found in wild-type and *Tlr2*^-/-^) were associated with stress and immune responses (S4 Fig). TLR2-independent pathways against *T*. *gondii* are known in some cells types, including macrophages and dendritic cells. For example, *T*. *gondii*-induced IL-12 production by macrophages and dendritic cells was impaired by the deficiency of myeloid differentiation primary response gene 88 (MyD88), an important adaptor molecule for most TLR signaling, but not by the deficiency of either TLR2 or TLR4 [63]. For downregulated genes, terms related to cellular responses to stimuli, signal transduction, and cell motility ranked in the top 20 (Fig 4D).

Genes upregulated in wild-type macrophages but not in *Tlr2*^-/-^ included some C-C chemokines (*Ccl2*, *Ccl7*) and genes related to stress and immune responses (*Slpi*, *Batf*, *Gsto1*, *Pf4*) (Fig 4E and 4F, S8 and S9 Tables). CCL2 and CCL7 are closely related chemokines, both of which attract monocytes. This suggests that the chemotactic activity of macrophages is TLR2-dependent. Moreover, these chemokines are reportedly regulated by TLR2 in microglia [42]. In our results, fold-changes for both *Ccl2* and *Ccl7* were higher in wild-type microglia (2.0 and 4.3, respectively) than in *Tlr2*^-/-^ (1.1 and 2.5, respectively). However, there were also many genes for which a relationship with the immune system is unknown. For example, *Ms4a6d*, encoding membrane-spanning 4-domains subfamily A member 6D, was highly upregulated only in wild-type macrophages (S8 Table), but there has been no report on the function of this gene in immune responses. *Cyb5r1*, encoding NADH-cytochrome b5 reductase 1, also has no previously reported relationship with immune responses. This gene is involved in desaturation and elongation of fatty acids, cholesterol biosynthesis, drug metabolism, and (in erythrocytes) methemoglobin reduction, as inferred from sequence similarity. TLR2-dependent downregulated genes also included some immune-related genes (*C1qb*, *Cd5l*, *Ciita*, *Vsig4*), as well as genes related to genome stability (*Dnmt3a*, *Zranb3*), and extracellular matrix and cell binding (*Col6a1*, *Col6a3*, *Jup*, *Mmp19*) (Fig 4F, S9 Table).

### Comparison of expression profiles among brain cells

**Fig 5:**
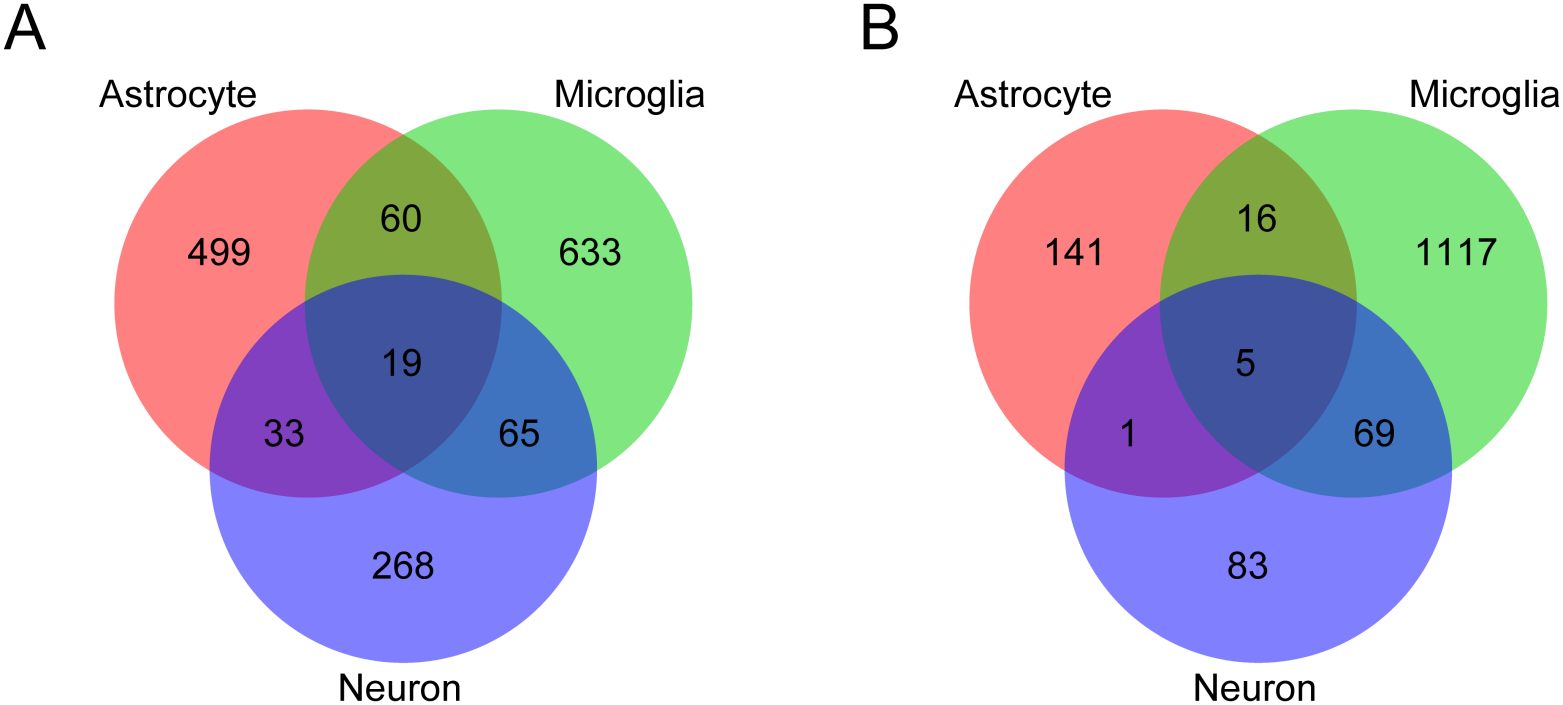
Comparison of TLR2-dependent genes among different CNS cell types. Venn diagrams comparing TLR2-dependent DEGs in *T*. *gondii* infection among astrocytes, microglia, and neurons. A, upregulated; B, downregulated.

Venn diagrams were created to identify TLR2-dependent DEGs that were specifically or commonly upregulated or downregulated in different CNS cell types during *T*. *gondii* infection (Fig 5A and 5B). The number of genes upregulated specifically in each cell type was 499 for astrocytes, 633 for microglia, and 268 for neurons (Fig 5A). In contrast, only 19 genes were upregulated commonly in all three cell types. The number of genes downregulated specifically in each cell type was 141 for astrocytes, 1117 for microglia, and 83 for neurons (Fig 5B), while only five genes were downregulated in all three cell types. TLR2-dependent DEGs commonly upregulated in all three cell types were mainly associated with immune responses (*Ccl3*, *Ets1*, *Csf3*, *Pvr*, *Cd14*, *Tlr1*), cell migration (*Ccl3*, *Mmp9*, *Stap1*, *Ets1*, *Serpine1*), inflammatory responses (*Ccl3*, *Tnip1*, *Chil3l1*, *Ptges*), and the TLR signaling pathway (*Tnip1*, *Cd14*, *Tlr1*) (Table 1). This may suggest that TLR2 is an important regulator of these immune-related genes in all brain cells examined during infection. Downregulated genes were associated with signal transduction (*Adcyap1r1*, *Plxnb3*, *Slc39a12*) and ion transport (*Slc39a12*, *Kcna6*) (Table 2), suggesting interaction between the TLR2 pathway and *T*. *gondii* infection is commonly related to ion channel-mediated signal transduction in all three CNS cell types.

**Table 1:** TLR2-dependent DEGs upregulated commonly in brain cells.

Gene ID	Symbol	Full name
ENSMUSG00000000982	*Ccl3*	C-C Motif Chemokine Ligand 3
ENSMUSG00000017737	*Mmp9*	matrix metalloproteinase 9
ENSMUSG00000020205	*Phlda1*	pleckstrin homology like domain family A member 1
ENSMUSG00000020400	*Tnip1*	TNFAIP3 interacting protein 1
ENSMUSG00000024743	*Syt7*	synaptotagmin 7
ENSMUSG00000024981	*Acsl5*	acyl-CoA synthetase long-chain family member 5
ENSMUSG00000026942	*Traf2*	TNF receptor associated factor 2
ENSMUSG00000029254	*Stap1*	signal transducing adaptor family member 1
ENSMUSG00000032035	*Ets1*	ETS proto-oncogene 1, transcription factor
ENSMUSG00000033227	*Wnt6*	wingless-type MMTV integration site family, member 6
ENSMUSG00000037411	*Serpine1*	serpin family E member 1
ENSMUSG00000038067	*Csf3*	colony stimulating factor 3
ENSMUSG00000039519	*Cyp7b1*	cytochrome P450 family 7 subfamily B member 1
ENSMUSG00000040511	*Pvr*	poliovirus receptor
ENSMUSG00000044827	*Tlr1*	toll like receptor 1
ENSMUSG00000050010	*Shisa3*	shisa family member 3
ENSMUSG00000050737	*Ptges*	prostaglandin E synthase
ENSMUSG00000051439	*Cd14*	CD14 molecule
ENSMUSG00000064246	*Chi3l1*	chitinase 3 like 1

**Table 2:** TLR2-dependent DEGs downregulated commonly in brain cells.

Gene ID	Symbol	Full name
ENSMUSG00000026227	*2810459M11Rik*	RIKEN cDNA 2810459M11 gene
ENSMUSG00000029778	*Adcyap1r1*	adenylate cyclase activating polypeptide 1 receptor 1
ENSMUSG00000031385	*Plxnb3*	plexin B3
ENSMUSG00000036949	*Slc39a12*	solute carrier family 39 member 12
ENSMUSG00000038077	*Kcna6*	potassium voltage-gated channel, shaker-related, subfamily, member 6

**Fig 6:**
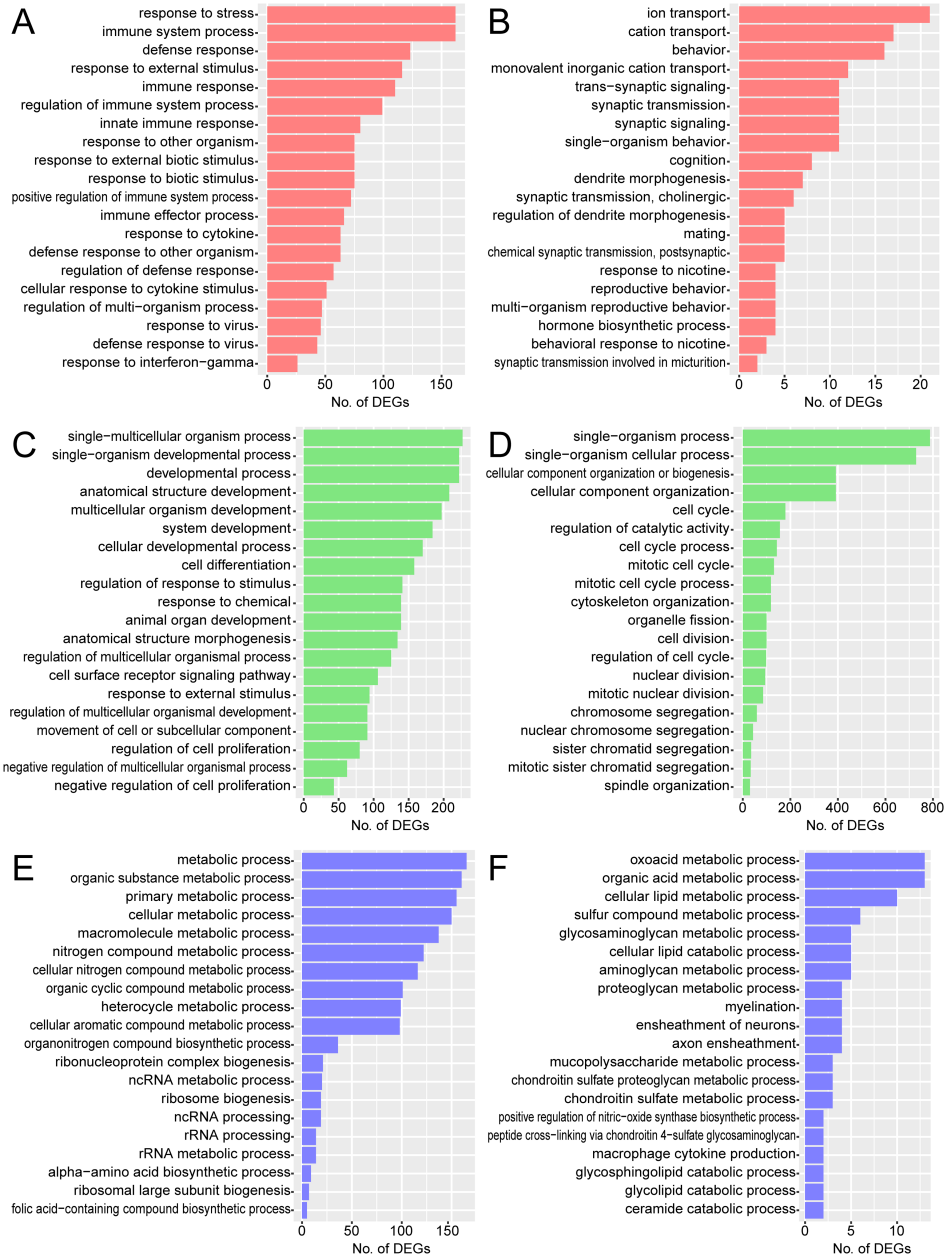
GO analysis of TLR2-dependent DEGs in different CNS cell types. GO analysis was performed to overview the functions of TLR2-dependent DEGs upregulated or downregulated specifically in astrocytes (A, B), microglia (C, D), or neurons (E, F) during *T*. *gondii* infection. Top 20 GO terms associated with biological process are listed. A, C, and E, upregulated; B, D and F, downregulated.

GO analysis was performed to overview the function of the TLR2-dependent genes specifically or commonly expressed in different types of brain cells (Fig 6). For astrocytes, overrepresented GO terms for upregulated genes were associated with immune and stress responses, and cytokines, similar to results not considering cell type (Fig 6A). For downregulated genes, GO terms were associated with behavior and ion transport (Fig 6B). Considering the large population of astrocytes in the brain, this result may suggest specific and major roles for astrocytes in the immune response against *T*. *gondii* in the brain. Moreover, *T*. *gondii* infection may inhibit normal functions of astrocytes related to ion transport for neurons, leading to behavioral disorders in host animals. For microglia, overrepresented GO terms included cell differentiation, movement of cell or subcellular components, negative regulation of cell proliferation, as well as responses to stimuli, while cell cycle and cell division were downregulated (Fig 6C and 6D). This result may reflect activation of microglia by TLR2-dependent responses to *T*. *gondii* infection, as the proliferation of these motile brain phagocytes is also regulated during infection. For neurons, GO terms overrepresented for both upregulated and downregulated genes showed various metabolic processes, while immune response dropped from the top 20 (Fig 6E and 6F), suggesting TLR2 is related to metabolic processes, but does not have a major role in immune responses within neurons. The ratio of total raw read counts for *T*. *gondii* transcripts to total counts for mouse transcripts was different among different brain cell types. This might be because of the difference in the efficiency of invasion of *T*. *gondii* tachyzoites into host cells and subsequent proliferation as well as different multiplicity of infection and difference in normal transcription levels of total RNA in each cell type (S5 Fig). In particular, *Tlr2*^-/-^ microglia showed a significantly higher ratio than wild-type, suggesting the TLR2-dependent microbicidal activity. Different invasion and proliferation efficiency may secondarily affect different expression profiles among cell types and between genotypes. In a further study, these points should also be considered when considering effects of cell types and genotypes on gene expression profiles.

### Comparison of expression profiles between phagocytic cells

**Fig 7:**
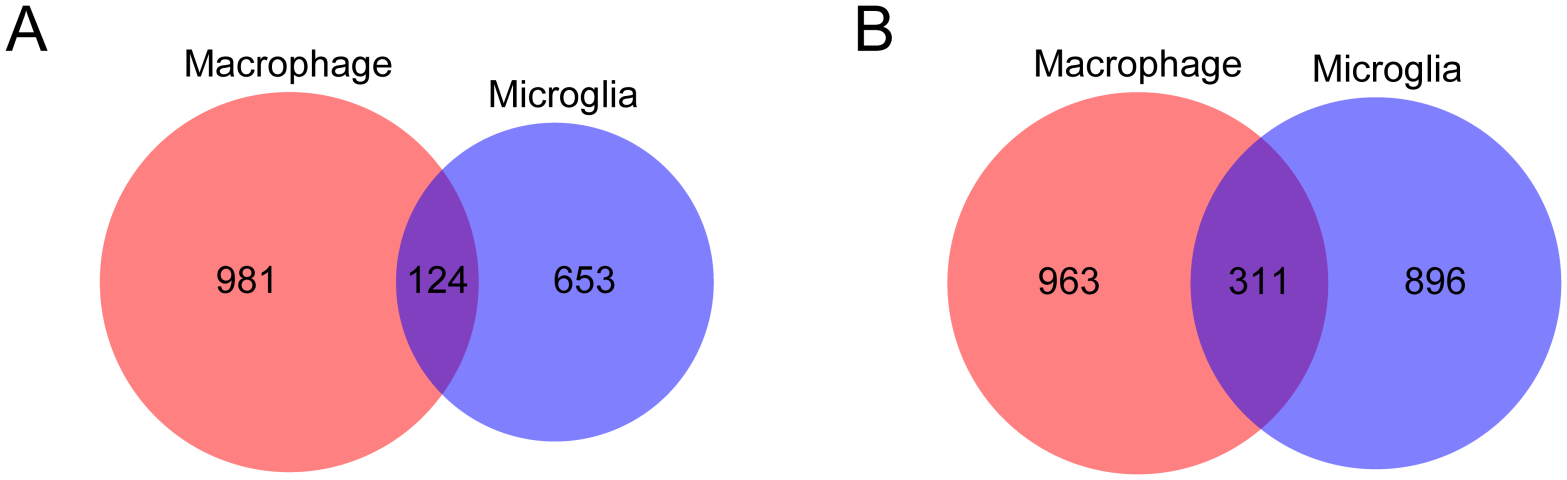
Comparison of TLR2-dependent genes between different phagocytic cell types. Venn diagrams were created to compare TLR2-dependent DEGs in *T*. *gondii* infection between macrophages and microglia. A, upregulated; B, downregulated.

TLR2-dependent DEGs were compared between two different types of phagocytic cells: macrophages and microglia (Fig 7). The number of genes specifically upregulated in each cell type were 981 for macrophages and 653 for microglia. In contrast, the number of genes upregulated in both cell types was only 124. The number of genes specifically downregulated in each cell type was 963 for macrophages and 896 for microglia, while the number of genes downregulated in both cell types was 311.

**Fig 8:**
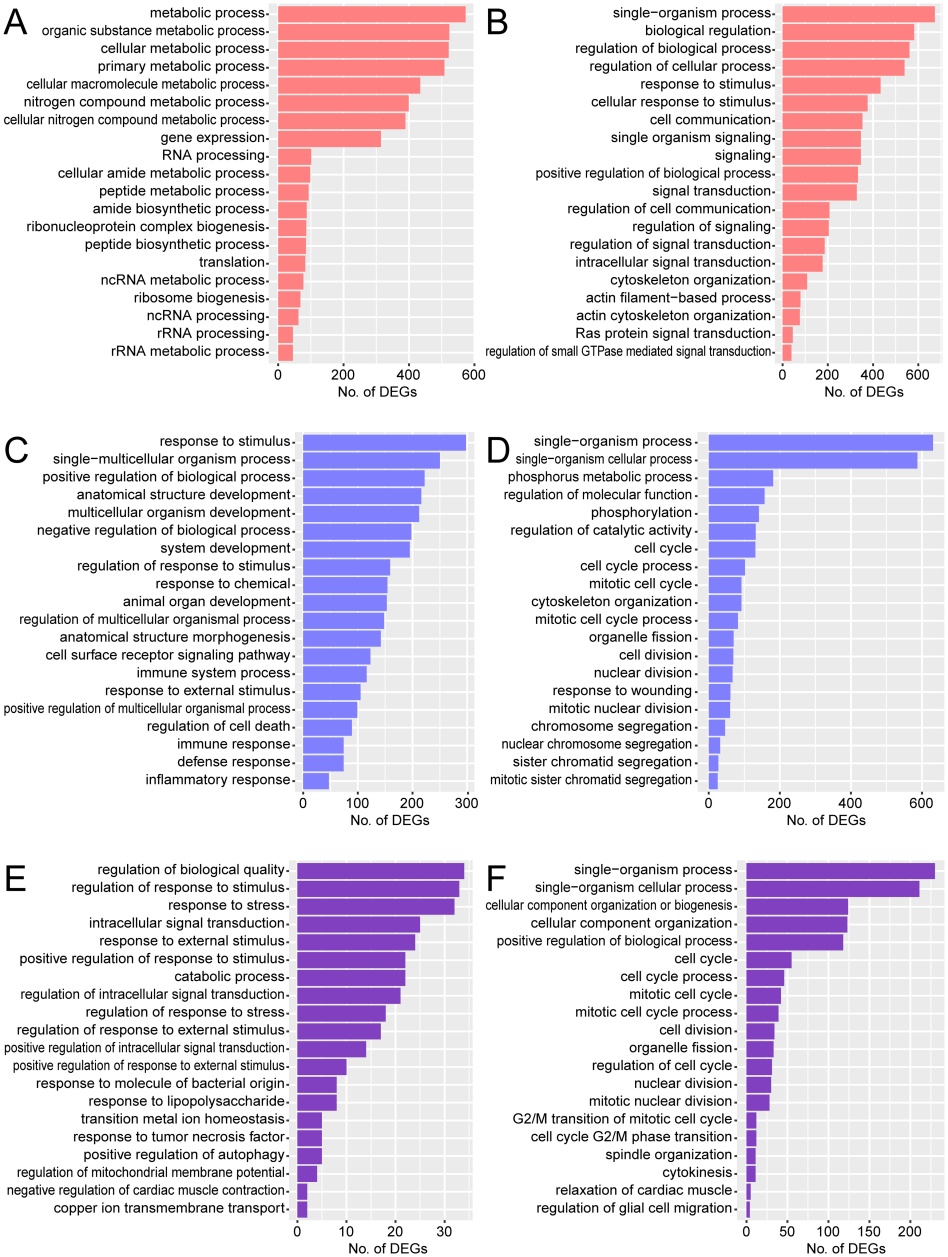
GO analysis of TLR2-dependent DEGs in different phagocytic cell types. GO analysis was performed to overview the functions of TLR2-dependent DEGs upregulated or downregulated specifically in macrophages (A, B), microglia (C, D), or commonly in both cell types (E, F) during *T*. *gondii* infection. Top 20 GO terms associated with biological process are listed. A, C, and E, upregulated; B, D, and F, downregulated.

GO analysis was performed to overview the function of TLR2-dependent genes specifically or commonly expressed in different phagocytic cell types (Fig 8). GO analysis of upregulated genes showed that metabolic process-related terms were overrepresented for macrophages, while immune response-related terms were overrepresented for microglia (Fig 8A and 8C). Responses to stress and stimuli were overrepresented for TLR2-dependent genes commonly in both phagocytic cell types (Fig 8E). In contrast, downregulated genes were related to response to stimuli and signaling for macrophages, but cell cycle and cell division for microglia (Fig 8B and 8D). Genes related to cell cycle and cell division were downregulated in both cell types (Fig 8F). Macrophages and microglia both contribute to the immune response as phagocytic and antigen-presenting cells, and both promote inflammation by secreting cytokines [64]. However, microglia have additional characteristics differing from macrophages, such as tightly regulated spatiality and low turnover rate. Our results suggested that, via different gene expression pathways, TLR2 exerted different functions and importance for microglia and macrophages during *T*. *gondii* infection. Both phagocytic cells showed a significantly higher ratio of total raw read counts for *T*. *gondii* transcripts to total counts for mouse transcripts in *Tlr2*^-/-^ than in wild-type, suggesting the TLR2-dependent microbicidal activity of these cells (S5 Fig).

### In vitro analysis of immune responses after *T*. *gondii* infection

**Fig 9:**
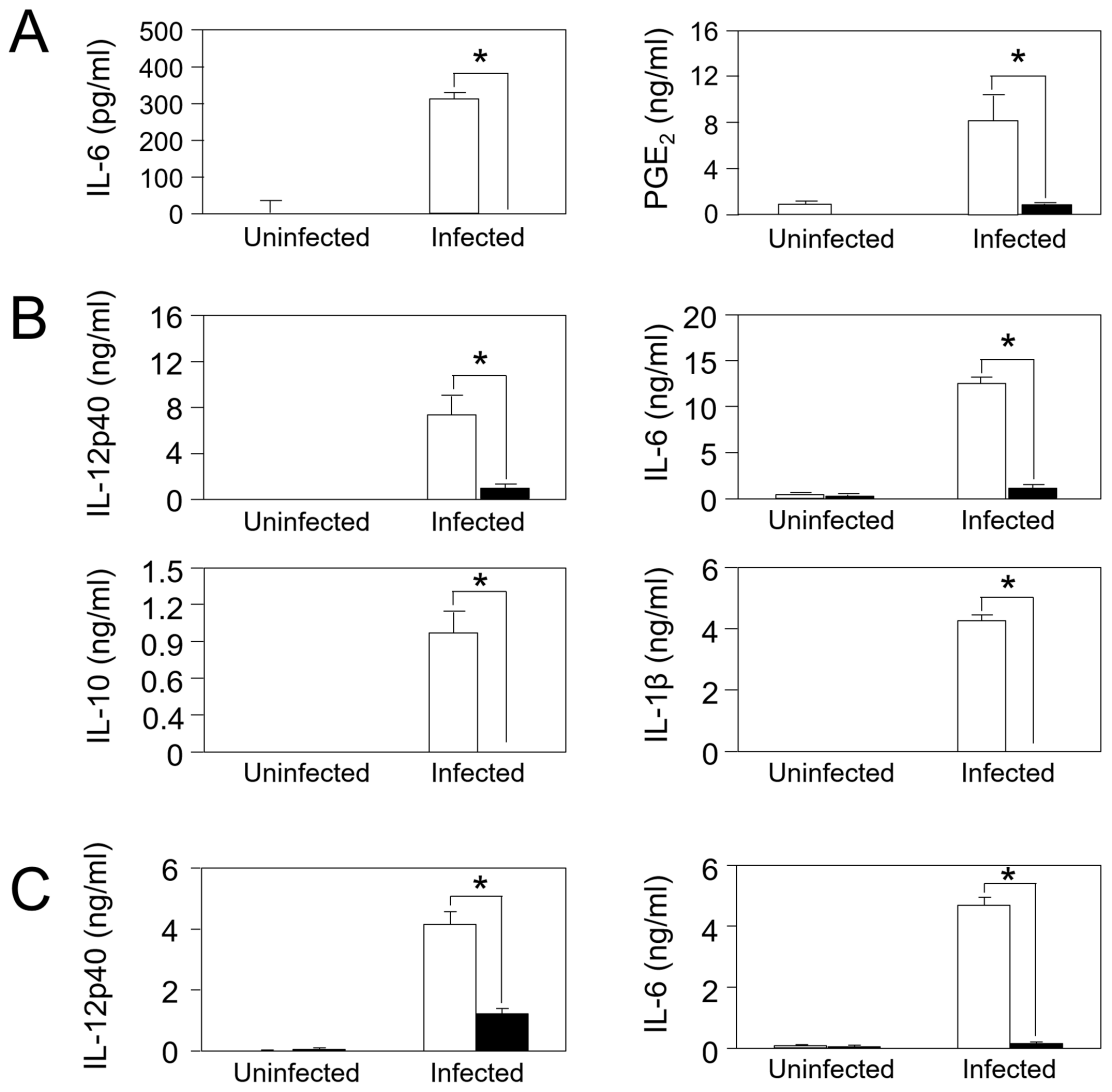
Production of cytokines and PGE_2_ by astrocytes, microglia, and peritoneal macrophages after *T*. *gondii* infection. Primary astrocytes (A), microglia (B), and peritoneal macrophages (C) from wild-type (white) and *Tlr2*^-/-^ mice (black) were infected with *T*. *gondii* tachyzoites. Each bar represents the mean ± SD of triplicate wells for each group. This is a representative result of two independent experiments. Asterisks represent significant differences with *p* < 0.05 in Student’s t-test.

The effects of deficiency of TLR2 on immunological pathways were examined in vitro. *Tlr2*^-/-^ astrocytes showed significantly lower production of IL-6 and PGE_2_ than wild-type ones after *T*. *gondii* infection (Fig 9A), corresponding to gene expression levels of *Il6* and *Ptges* (encoding prostaglandin E synthase; S10 Table). Wild-type microglia responded to *T*. *gondii* infection with the production of cytokines such as IL-12p40, IL-6, IL-10, and IL-1β, while *Tlr2*^-/-^ cells showed much or completely impaired production of these cytokines (Fig 9B). These results are corresponding to much less expression of the genes encoding these cytokines in *Tlr2*^-/-^ microglia compared to wild-type (S11 Table). Peritoneal macrophages also showed similar results for the production and gene expression of IL-6 and IL-12p40 (Fig 9C; S12 Table). These results demonstrated that these immune-related and neuroprotective factors are not only expressed as genes but also are secreted as proteins in a TLR2-dependent manner.

**Fig 10:**
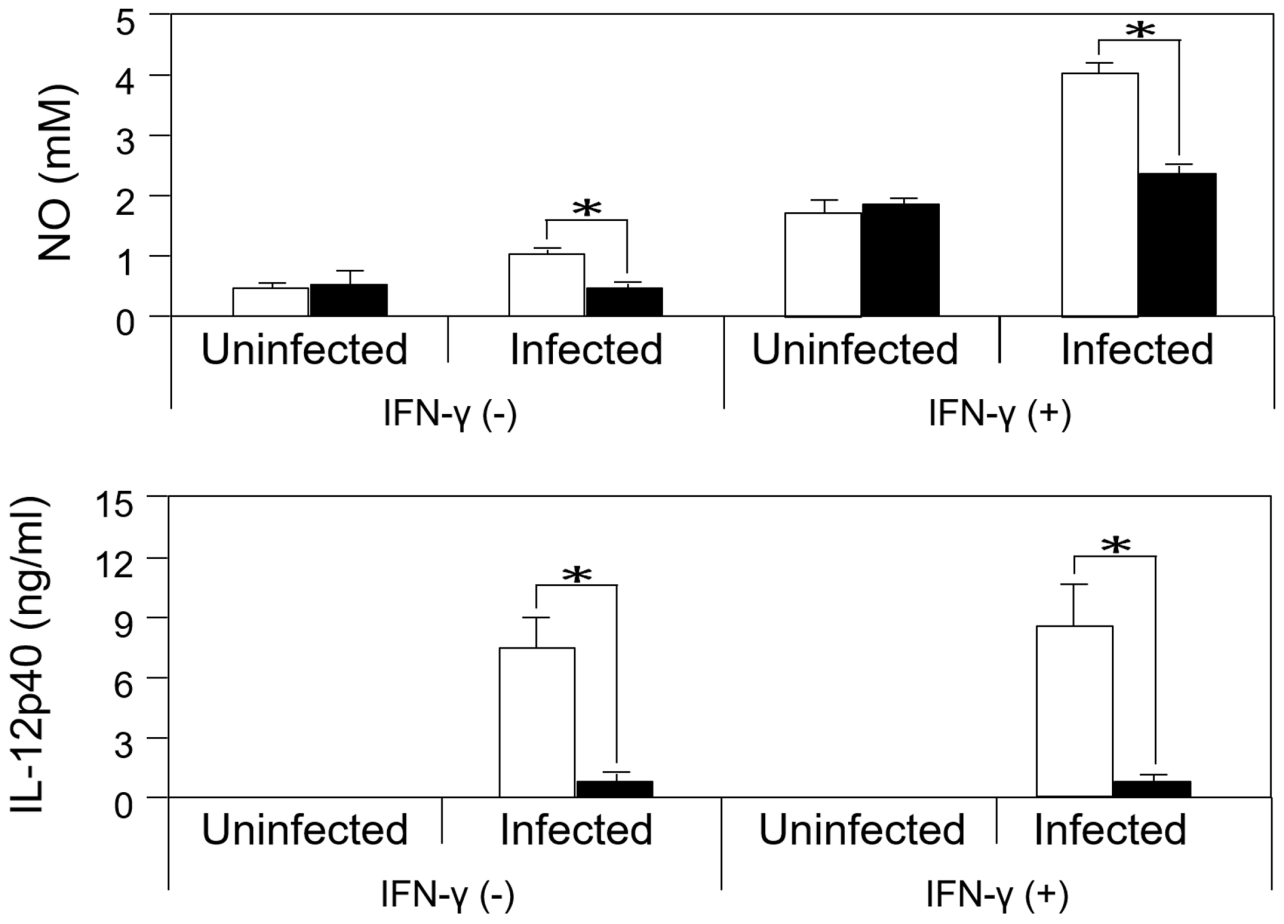
Production of NO and IL-12p40 from microglia stimulated with IFN-γ. Microglia from wild-type (white) and *Tlr2*^-/-^ mice (black) were infected with *T*. *gondii* tachyzoites. Each bar represents the mean ± SD of triplicate wells for each group. This is a representative result of two independent experiments. Asterisks represent significant differences with *p* < 0.05 in Student’s t-test.

The production of NO, an important molecule for the clearance of *T*. *gondii*, was measured for microglial cells after *T*. *gondii* infection and IFN-γ stimulation in order to examine whether the production is dependent on TLR2. *T*. *gondii* infection significantly increased NO production in wild-type microglia, but not in *Tlr2*^-/-^ cells (Fig 10). IL-12p40 production was also measured under stimulation with IFN-γ. The production was significantly higher in infected wild-type microglia than in infected *Tlr2*^-/-^ cells but not affected by IFN-γ (Fig 10). These results likely reflected much higher gene expression in wild-type than in *Tlr2*^-/-^ (S11 Table) and indicated that microglia produce NO and IL-12p40 in a TLR2-dependent manner.

### Conclusions

This is the first report of TLR2-dependent gene expression profiling during *T*. *gondii* infection that compares different CNS cell types. Our results showed that TLR2 regulates many genes in the brain during *T*. *gondii* infection, and that TLR2-dependent genes vary greatly between different CNS cell types. Importantly, genes identified included many whose host immune system functions have been reported in previous studies, confirming the consistency and reliability of our results. In vitro experiments for levels of immune-related molecules also confirmed the importance of TLR2 in immune responses against *T*. *gondii*. Moreover, many genes whose function within the immune system remains unknown were also upregulated or downregulated in a TLR2-dependent manner. It is still unclear whether differential expression of these genes is required for *T*. *gondii* to maintain a relationship with the host or required for the host to maintain brain homeostasis by elimination of the parasite. Although further study is needed to reveal the unknown functions of genes regulated by TLR2, our study provides important basic information about TLR2-regulated host-parasite interactions within the brain, and will facilitate full elucidation of the relationship between *T*. *gondii* infection and the host immune system.

## Supporting information

S1 Fig Identification of purity of primary astrocytes. Cells positively stained for glial fibrillary acidic protein were identified as astrocytes by immunofluorescence microscopy (A, C). Flow cytometry confirmed that few of them were positively stained with phycoerythrin (PE) labeled anti-mouse CD11b antibody (y axis; B, D). A and B, wild-type; C and D, *Tlr2*^-/-^.
(TIF)

S2 Fig Identification of purity of primary microglia. Cells positively stained with phycoerythrin (PE) labeled anti-mouse CD11b antibody (y axis) were identified as microglia by flow cytometry. A, wild-type; B, *Tlr2*^-/-^.
(TIF)

S3 Fig Identification of purity of peritoneal macrophages. Cells positively stained with phycoerythrin (PE) labeled anti-mouse CD11b antibody (x axis) were identified as macrophages by flow cytometry. A, wild-type; B, *Tlr2*^-/-^.
(TIF)

S4 Fig Top 20 GO terms overrepresented for DEGs TLR2-independently upregulated in macrophages (upregulated in both wild-type and *Tlr2*^-/-^). WT, wild-type; KO, *Tlr2*^-/-^. Asterisks represent significant differences with *p* < 0.05 in Fisher’s exact test.
(TIF)

S5 Fig Comparison of infection efficiency between genotypes. Ratio of raw read counts for *T*. *gondii* transcripts to counts for mouse transcripts were compared between wild-type and *Tlr2*^-/-^ in each cell type. Asterisks represent significant differences with *p* < 0.05 in Student’s t-test after arcsine transformation.
(TIF)

S1 Table Detailed expression data for top 20 DEGs TLR2-dependently upregulated in astrocytes. (XLSX)

S2 Table Detailed expression data for top 20 DEGs TLR2-dependently downregulated in astrocytes. (XLSX)

S3 Table Expression data for IFNs in astrocytes. (XLSX)

S4 Table Detailed expression data for top 20 DEGs TLR2-dependently upregulated in microglia. (XLSX)

S5 Table Detailed expression data for top 20 DEGs TLR2-dependently downregulated in microglia. (XLSX)

S6 Table Detailed expression data for top 20 DEGs TLR2-dependently upregulated in neurons. (XLSX)

S7 Table Detailed expression data for top 20 DEGs TLR2-dependently downregulated in neurons. (XLSX)

S8 Table Detailed expression data for top 20 DEGs TLR2-dependently upregulated in macrophages. (XLSX)

S9 Table Detailed expression data for top 20 DEGs TLR2-dependently downregulated in macrophages. (XLSX)

S10 Table Expression data for *Il6* and prostaglandin E synthases in astrocytes. (XLSX)

S11 Table Expression data for cytokines and *Nos2* in microglia. (XLSX)

S12 Table Expression data for *Il6* and *Il12b* in peritoneal macrophages. (XLSX)

## References

[1] Montoya J, Liesenfeld O. Toxoplasmosis. The Lancet. 2004;363: 1965–1976. doi: 10.1016/S0140-6736(04)16412-X 10.1016/S0140-6736(04)16412-X 15194258

[2] Luft BJ, Conley F, Remington JS, Laverdiere M, Wagner KF, Levine JF, et al Outbreak of central-nervous-system toxoplasmosis in western Europe and North America. Lancet Lond Engl. 1983;1: 781–784. 10.1016/s0140-6736(83)91847-0 6132129

[3] Jones J, Lopez A, Wilson M. Congenital toxoplasmosis. Am Fam Physician. 2003;67: 2131–2138. 12776962

[4] Black MW, Boothroyd JC. Lytic Cycle of *Toxoplasma gondii*. Microbiol Mol Biol Rev. 2000;64: 607–623. 10974128 10.1128/mmbr.64.3.607-623.2000 PMC99006

[5] Skariah S, McIntyre MK, Mordue DG. *Toxoplasma gondii*: determinants of tachyzoite to bradyzoite conversion. Parasitol Res. 2010;107: 253–260. doi: 10.1007/s00436-010-1899-6 20514494 10.1007/s00436-010-1899-6 PMC3327608

[6] Weiss LM, Kim K. The development and biology of bradyzoites of *Toxoplasma gondii*. Front Biosci J Virtual Libr. 2000;5: D391–405. 10.2741/weiss PMC3109641 10762601

[7] Hermes G, Ajioka JW, Kelly KA, Mui E, Roberts F, Kasza K, et al Neurological and behavioral abnormalities, ventricular dilatation, altered cellular functions, inflammation, and neuronal injury in brains of mice due to common, persistent, parasitic infection. J Neuroinflammation. 2008;5: 48 doi: 10.1186/1742-2094-5-48 18947414 10.1186/1742-2094-5-48 PMC2588578

[8] Lüder CGK, Giraldo-Velásquez M, Sendtner M, Gross U. *Toxoplasma gondii* in Primary Rat CNS Cells: Differential Contribution of Neurons, Astrocytes, and Microglial Cells for the Intracerebral Development and Stage Differentiation. Exp Parasitol. 1999;93: 23–32. doi: 10.1006/expr.1999.4421 10464035 10.1006/expr.1999.4421

[9] Cabral CM, Tuladhar S, Dietrich HK, Nguyen E, MacDonald WR, Trivedi T, et al Neurons are the Primary Target Cell for the Brain-Tropic Intracellular Parasite *Toxoplasma gondii*. PLOS Pathog. 2016;12: e1005447 doi: 10.1371/journal.ppat.1005447 26895155 10.1371/journal.ppat.1005447 PMC4760770

[10] Melzer TC, Cranston HJ, Weiss LM, Halonen SK. Host Cell Preference of *Toxoplasma gondii* Cysts in Murine Brain: A Confocal Study. J Neuroparasitology. 2010;1 doi: 10.4303/jnp/N100505 21625284 10.4303/jnp/N100505 PMC3103221

[11] Contreras-Ochoa CO, Lagunas-Martínez A, Belkind-Gerson J, Correa D. *Toxoplasma gondii* invasion and replication in astrocyte primary cultures and astrocytoma cell lines: systematic review of the literature. Parasitol Res. 2012;110: 2089–2094. doi: 10.1007/s00436-012-2836-7 22314782 10.1007/s00436-012-2836-7

[12] Kreutzberg GW. Microglia: a sensor for pathological events in the CNS. Trends Neurosci. 1996;19: 312–318. doi: 10.1016/0166-2236(96)10049-7 8843599 10.1016/0166-2236(96)10049-7

[13] Dellacasa-Lindberg I, Fuks JM, Arrighi RBG, Lambert H, Wallin RPA, Chambers BJ, et al Migratory Activation of Primary Cortical Microglia upon Infection with *Toxoplasma gondii*. Infect Immun. 2011;79: 3046–3052. doi: 10.1128/IAI.01042-10 21628522 10.1128/IAI.01042-10 PMC3147544

[14] Sanecka A, Frickel E-M. Use and abuse of dendritic cells by *Toxoplasma gondii*. Virulence. 2012;3: 678–689. doi: 10.4161/viru.22833 23221473 10.4161/viru.22833 PMC3545950

[15] Takeuchi O, Hoshino K, Akira S. Cutting Edge: TLR2-Deficient and MyD88-Deficient Mice Are Highly Susceptible to *Staphylococcus aureus* Infection. J Immunol. 2000;165: 5392–5396. doi: 10.4049/jimmunol.165.10.5392 11067888 10.4049/jimmunol.165.10.5392

[16] Thoma-Uszynski S, Stenger S, Takeuchi O, Ochoa MT, Engele M, Sieling PA, et al Induction of Direct Antimicrobial Activity Through Mammalian Toll-Like Receptors. Science. 2001;291: 1544–1547. doi: 10.1126/science.291.5508.1544 11222859 10.1126/science.291.5508.1544

[17] Mun HS, Aosai F, Norose K, Chen M, Piao LX, Takeuchi O, et al TLR2 as an essential molecule for protective immunity against *Toxoplasma gondii* infection. Int Immunol. 2003;15: 1081–1087. doi: 10.1093/intimm/dxg108 12917260 10.1093/intimm/dxg108

[18] Debierre-Grockiego F, Campos MA, Azzouz N, Schmidt J, Bieker U, Resende MG, et al Activation of TLR2 and TLR4 by Glycosylphosphatidylinositols Derived from *Toxoplasma gondii*. J Immunol. 2007;179: 1129–1137. doi: 10.4049/jimmunol.179.2.1129 17617606 10.4049/jimmunol.179.2.1129

[19] Crack PJ, Bray PJ. Toll-like receptors in the brain and their potential roles in neuropathology. Immunol Cell Biol. 2007;85: 476–480. doi: 10.1038/sj.icb.7100103 17667932 10.1038/sj.icb.7100103

[20] Fischer HG, Nitzgen B, Germann T, Degitz K, Däubener W, Hadding U. Differentiation driven by granulocyte-macrophage colony-stimulating factor endows microglia with interferon-gamma-independent antigen presentation function. J Neuroimmunol. 1993;42: 87–95. 8093703 10.1016/0165-5728(93)90215-k

[21] Hilgenberg LGW, Smith MA. Preparation of Dissociated Mouse Cortical Neuron Cultures. J Vis Exp JoVE. 2007; doi: 10.3791/562 18989405 10.3791/562 PMC2557074

[22] Tanaka S, Nishimura M, Ihara F, Yamagishi J, Suzuki Y, Nishikawa Y. Transcriptome Analysis of Mouse Brain Infected with *Toxoplasma gondii*. Infect Immun. 2013;81: 3609–3619. doi: 10.1128/IAI.00439-13 23856619 10.1128/IAI.00439-13 PMC3811780

[23] Gajria B, Bahl A, Brestelli J, Dommer J, Fischer S, Gao X, et al ToxoDB: an integrated *Toxoplasma gondii* database resource. Nucleic Acids Res. 2008;36: D553–D556. doi: 10.1093/nar/gkm981 18003657 10.1093/nar/gkm981 PMC2238934

[24] Anders S, Huber W. Differential expression analysis for sequence count data. Genome Biol. 2010;11: R106 doi: 10.1186/gb-2010-11-10-r106 20979621 10.1186/gb-2010-11-10-r106 PMC3218662

[25] Eppig JT, Blake JA, Bult CJ, Kadin JA, Richardson JE. The Mouse Genome Database (MGD): comprehensive resource for genetics and genomics of the laboratory mouse. Nucleic Acids Res. 2012;40: D881–D886. doi: 10.1093/nar/gkr974 22075990 10.1093/nar/gkr974 PMC3245042

[26] Young MD, Wakefield MJ, Smyth GK, Oshlack A. Gene ontology analysis for RNA-seq: accounting for selection bias. Genome Biol. 2010;11: R14 doi: 10.1186/gb-2010-11-2-r14 20132535 10.1186/gb-2010-11-2-r14 PMC2872874

[27] Yamamoto M, Okuyama M, Ma JS, Kimura T, Kamiyama N, Saiga H, et al A cluster of interferon-γ-inducible p65 GTPases plays a critical role in host defense against *Toxoplasma gondii*. Immunity. 2012;37: 302–313. doi: 10.1016/j.immuni.2012.06.009 22795875 10.1016/j.immuni.2012.06.009

[28] Degrandi D, Kravets E, Konermann C, Beuter-Gunia C, Klümpers V, Lahme S, et al Murine Guanylate Binding Protein 2 (mGBP2) controls *Toxoplasma gondii* replication. Proc Natl Acad Sci U S A. 2013;110: 294–299. doi: 10.1073/pnas.1205635110 23248289 10.1073/pnas.1205635110 PMC3538222

[29] Pittman KJ, Aliota MT, Knoll LJ. Dual transcriptional profiling of mice and *Toxoplasma gondii* during acute and chronic infection. BMC Genomics. 2014;15: 806 doi: 10.1186/1471-2164-15-806 25240600 10.1186/1471-2164-15-806 PMC4177681

[30] Taylor GA, Feng CG, Sher A. p47 GTPases: regulators of immunity to intracellular pathogens. Nat Rev Immunol. 2004;4: 100–109. doi: 10.1038/nri1270 15040583 10.1038/nri1270

[31] Martens S, Howard J. The interferon-inducible GTPases. Annu Rev Cell Dev Biol. 2006;22: 559–589. doi: 10.1146/annurev.cellbio.22.010305.104619 16824009 10.1146/annurev.cellbio.22.010305.104619

[32] Ghosh SK, Kusari J, Bandyopadhyay SK, Samanta H, Kumar R, Sen GC. Cloning, sequencing, and expression of two murine 2’-5’-oligoadenylate synthetases. Structure-function relationships. J Biol Chem. 1991;266: 15293–15299. 1651324

[33] Freshman MM, Merigan TC, Remington JS, Brownlee IE. In vitro and in vivo antiviral action of an interferon-like substance induced by *Toxoplasma gondii*. Proc Soc Exp Biol Med Soc Exp Biol Med N Y N. 1966;123: 862–866. 10.3181/00379727-123-31625 4289528

[34] Board PG, Losowsky MS, Miloszewski KJ. Factor XIII: inherited and acquired deficiency. Blood Rev. 1993;7: 229–242. 8130686 10.1016/0268-960x(93)90010-2

[35] Holst FG, Hemmer CJ, Foth C, Seitz R, Egbring R, Dietrich M. Low levels of fibrin-stabilizing factor (factor XIII) in human *Plasmodium falciparum* malaria: correlation with clinical severity. Am J Trop Med Hyg. 1999;60: 99–104. 9988331 10.4269/ajtmh.1999.60.99

[36] Sugitani K, Ogai K, Hitomi K, Nakamura-Yonehara K, Shintani T, Noda M, et al A distinct effect of transient and sustained upregulation of cellular factor XIII in the goldfish retina and optic nerve on optic nerve regeneration. Neurochem Int. 2012;61: 423–432. doi: 10.1016/j.neuint.2012.06.004 22709671 10.1016/j.neuint.2012.06.004

[37] Mariani MM, Kielian T. Microglia in Infectious Diseases of the Central Nervous System. J Neuroimmune Pharmacol Off J Soc NeuroImmune Pharmacol. 2009;4: 448–461. doi: 10.1007/s11481-009-9170-6 19728102 10.1007/s11481-009-9170-6 PMC2847353

[38] Sofroniew MV, Vinters HV. Astrocytes: biology and pathology. Acta Neuropathol (Berl). 2010;119: 7–35. doi: 10.1007/s00401-009-0619-8 20012068 10.1007/s00401-009-0619-8 PMC2799634

[39] Drögemüller K, Helmuth U, Brunn A, Sakowicz-Burkiewicz M, Gutmann DH, Mueller W, et al Astrocyte gp130 Expression Is Critical for the Control of *Toxoplasma* Encephalitis. J Immunol. 2008;181: 2683–2693. doi: 10.4049/jimmunol.181.4.2683 18684959 10.4049/jimmunol.181.4.2683

[40] Gómez-Nicola D, Fransen NL, Suzzi S, Perry VH. Regulation of Microglial Proliferation during Chronic Neurodegeneration. J Neurosci. 2013;33: 2481–2493. doi: 10.1523/JNEUROSCI.4440-12.2013 23392676 10.1523/JNEUROSCI.4440-12.2013 PMC6619184

[41] Stenzel W, Soltek S, Sanchez-Ruiz M, Akira S, Miletic H, Schlüter D, et al Both TLR2 and TLR4 Are Required for the Effective Immune Response in *Staphylococcus aureus*-Induced Experimental Murine Brain Abscess. Am J Pathol. 2008;172: 132–145. doi: 10.2353/ajpath.2008.070567 18165267 10.2353/ajpath.2008.070567 PMC2189630

[42] Aravalli RN, Hu S, Rowen TN, Palmquist JM, Lokensgard JR. Cutting Edge: TLR2-Mediated Proinflammatory Cytokine and Chemokine Production by Microglial Cells in Response to Herpes Simplex Virus. J Immunol. 2005;175: 4189–4193. doi: 10.4049/jimmunol.175.7.4189 16177057 10.4049/jimmunol.175.7.4189

[43] Wilhelmsen K, Mesa K, Prakash A, Xu F, Hellman J. Activation of Endothelial TLR2 by Bacterial Lipoprotein Upregulates Proteins Specific for the Neutrophil Response. Innate Immun. 2012;18: 602–616. doi: 10.1177/1753425911429336 22186927 10.1177/1753425911429336 PMC3444510

[44] Neuner R, Cousin H, McCusker C, Coyne M, Alfandari D. *Xenopus* ADAM19 is involved in neural, neural crest and muscle development. Mech Dev. 2009;126: 240–255. doi: 10.1016/j.mod.2008.10.010 19027850 10.1016/j.mod.2008.10.010 PMC2754070

[45] Sridharan J, Haremaki T, Jin Y, Teegala S, Weinstein DC. Xmab21l3 mediates dorsoventral patterning in *Xenopus laevis*. Mech Dev. 2012;129: 136–146. doi: 10.1016/j.mod.2012.05.002 22609272 10.1016/j.mod.2012.05.002 PMC3409299

[46] Seiradake E, Coles CH, Perestenko PV, Harlos K, McIlhinney RAJ, Aricescu AR, et al Structural basis for cell surface patterning through NetrinG-NGL interactions. EMBO J. 2011;30: 4479–4488. doi: 10.1038/emboj.2011.346 21946559 10.1038/emboj.2011.346 PMC3230378

[47] Angulo JA, Cadet JL, Woolley CS, Suber F, McEwen BS. Effect of chronic typical and atypical neuroleptic treatment on proenkephalin mRNA levels in the striatum and nucleus accumbens of the rat. J Neurochem. 1990;54: 1889–1894. 1971007 10.1111/j.1471-4159.1990.tb04887.x

[48] Bjørnsen LP, Hadera MG, Zhou Y, Danbolt NC, Sonnewald U. The GLT-1 (EAAT2; slc1a2) glutamate transporter is essential for glutamate homeostasis in the neocortex of the mouse. J Neurochem. 2014;128: 641–649. doi: 10.1111/jnc.12509 24224925 10.1111/jnc.12509

[49] Bacaj T, Wu D, Yang X, Morishita W, Zhou P, Xu W, et al Synaptotagmin-1 and -7 Trigger Synchronous and Asynchronous Phases of Neurotransmitter Release. Neuron. 2013;80: 947–959. doi: 10.1016/j.neuron.2013.10.026 24267651 10.1016/j.neuron.2013.10.026 PMC3888870

[50] Jin S, Kim JG, Park JW, Koch M, Horvath TL, Lee BJ. Hypothalamic TLR2 triggers sickness behavior via a microglia-neuronal axis. Sci Rep. 2016;6: srep29424. doi: 10.1038/srep29424 27405276 10.1038/srep29424 PMC4942617

[51] Conseil V, Soête M, Dubremetz JF. Serine Protease Inhibitors Block Invasion of Host Cells by *Toxoplasma gondii*. Antimicrob Agents Chemother. 1999;43: 1358–1361. 10348752 10.1128/aac.43.6.1358 PMC89278

[52] Hill RD, Gouffon JS, Saxton AM, Su C. Differential Gene Expression in Mice Infected with Distinct *Toxoplasma* Strains. Infect Immun. 2012;80: 968–974. doi: 10.1128/IAI.05421-11 22144491 10.1128/IAI.05421-11 PMC3294647

[53] Lagal V, Binder EM, Huynh MH, Kafsack BF, Harris PK, Diez R, et al *Toxoplasma gondii* Protease TgSUB1 is Required for Cell Surface Processing of Micronemal Adhesive Complexes and Efficient Adhesion of Tachyzoites. Cell Microbiol. 2010;12: 1792–1808. doi: 10.1111/j.1462-5822.2010.01509.x 20678172 10.1111/j.1462-5822.2010.01509.x PMC2997387

[54] Peng BW, Lin J, Zhang T. *Toxoplasma gondii* induces prostaglandin E2 synthesis in macrophages via signal pathways for calcium-dependent arachidonic acid production and PKC-dependent induction of cyclooxygenase-2. Parasitol Res. 2008;102: 1043–1050. doi: 10.1007/s00436-007-0873-4 18305957 10.1007/s00436-007-0873-4

[55] Cho H, Huang L, Hamza A, Gao D, Zhan CG, Tai HH. Role of glutamine 148 of human 15-hydroxyprostaglandin dehydrogenase in catalytic oxidation of prostaglandin E2. Bioorg Med Chem. 2006;14: 6486–6491. doi: 10.1016/j.bmc.2006.06.030 16828555 10.1016/j.bmc.2006.06.030

[56] Gorfu G, Cirelli KM, Melo MB, Mayer-Barber K, Crown D, Koller BH, et al Dual Role for Inflammasome Sensors NLRP1 and NLRP3 in Murine Resistance to *Toxoplasma gondii*. mBio. 2014;5: e01117–13. doi: 10.1128/mBio.01117-13 24549849 10.1128/mBio.01117-13 PMC3944820

[57] Yang J, Goetz D, Li JY, Wang W, Mori K, Setlik D, et al An Iron Delivery Pathway Mediated by a Lipocalin. Mol Cell. 2002;10: 1045–1056. doi: 10.1016/S1097-2765(02)00710-4 12453413 10.1016/s1097-2765(02)00710-4

[58] Zhao H, Konishi A, Fujita Y, Yagi M, Ohata K, Aoshi T, et al Lipocalin 2 bolsters innate and adaptive immune responses to blood-stage malaria infection by reinforcing host iron metabolism. Cell Host Microbe. 2012;12: 705–716. doi: 10.1016/j.chom.2012.10.010 23159059 10.1016/j.chom.2012.10.010

[59] Oosterhoff JK, Penninkhof F, Brinkmann AO, Anton Grootegoed J, Blok LJ. REPS2/POB1 is downregulated during human prostate cancer progression and inhibits growth factor signalling in prostate cancer cells. Oncogene. 2003;22: 2920–2925. doi: 10.1038/sj.onc.1206397 12771942 10.1038/sj.onc.1206397

[60] Yoshida E, Atkinson TG, Chakravarthy B. Neuroprotective gene expression profiles in ischemic cortical cultures preconditioned with IGF-1 or bFGF. Mol Brain Res. 2004;131: 33–50. doi: 10.1016/j.molbrainres.2004.08.023 15530650 10.1016/j.molbrainres.2004.08.023

[61] Pasterkamp RJ, Kolk SM, Hellemons AJ, Kolodkin AL. Expression patterns of semaphorin7A and plexinC1during rat neural development suggest roles in axon guidance and neuronal migration. BMC Dev Biol. 2007;7: 98 doi: 10.1186/1471-213X-7-98 17727705 10.1186/1471-213X-7-98 PMC2008261

[62] Hu H, Marton TF, Goodman CS. Plexin B Mediates Axon Guidance in *Drosophila* by Simultaneously Inhibiting Active Rac and Enhancing RhoA Signaling. Neuron. 2001;32: 39–51. doi: 10.1016/S0896-6273(01)00453-6 11604137 10.1016/s0896-6273(01)00453-6

[63] Tang SC, Arumugam TV, Xu X, Cheng A, Mughal MR, Jo DG, et al Pivotal role for neuronal Toll-like receptors in ischemic brain injury and functional deficits. Proc Natl Acad Sci U S A. 2007;104: 13798–13803. doi: 10.1073/pnas.0702553104 17693552 10.1073/pnas.0702553104 PMC1959462

[64] Perry VH, Teeling J. Microglia and macrophages of the central nervous system: the contribution of microglia priming and systemic inflammation to chronic neurodegeneration. Semin Immunopathol. 2013;35: 601–612. doi: 10.1007/s00281-013-0382-8 23732506 10.1007/s00281-013-0382-8 PMC3742955

