# Correction: Transcriptional profiling of Toll-like receptor 2-deficient primary murine brain cells during *Toxoplasma gondii* infection

**DOI:** 10.1371/journal.pone.0303453

**Published:** 2024-05-02

**Authors:** Kousuke Umeda, Sachi Tanaka, Fumiaki Ihara, Junya Yamagishi, Yutaka Suzuki, Yoshifumi Nishikawa

The Fig 3E and 3F and Fig 4E and 4F are swapped in the Fig 3 and Fig 4. The current Fig 3E and 3F should be Fig 4E and 4F and vice versa. Please see the correct Fig 3 and Fig 4 here.

**Fig 3:**
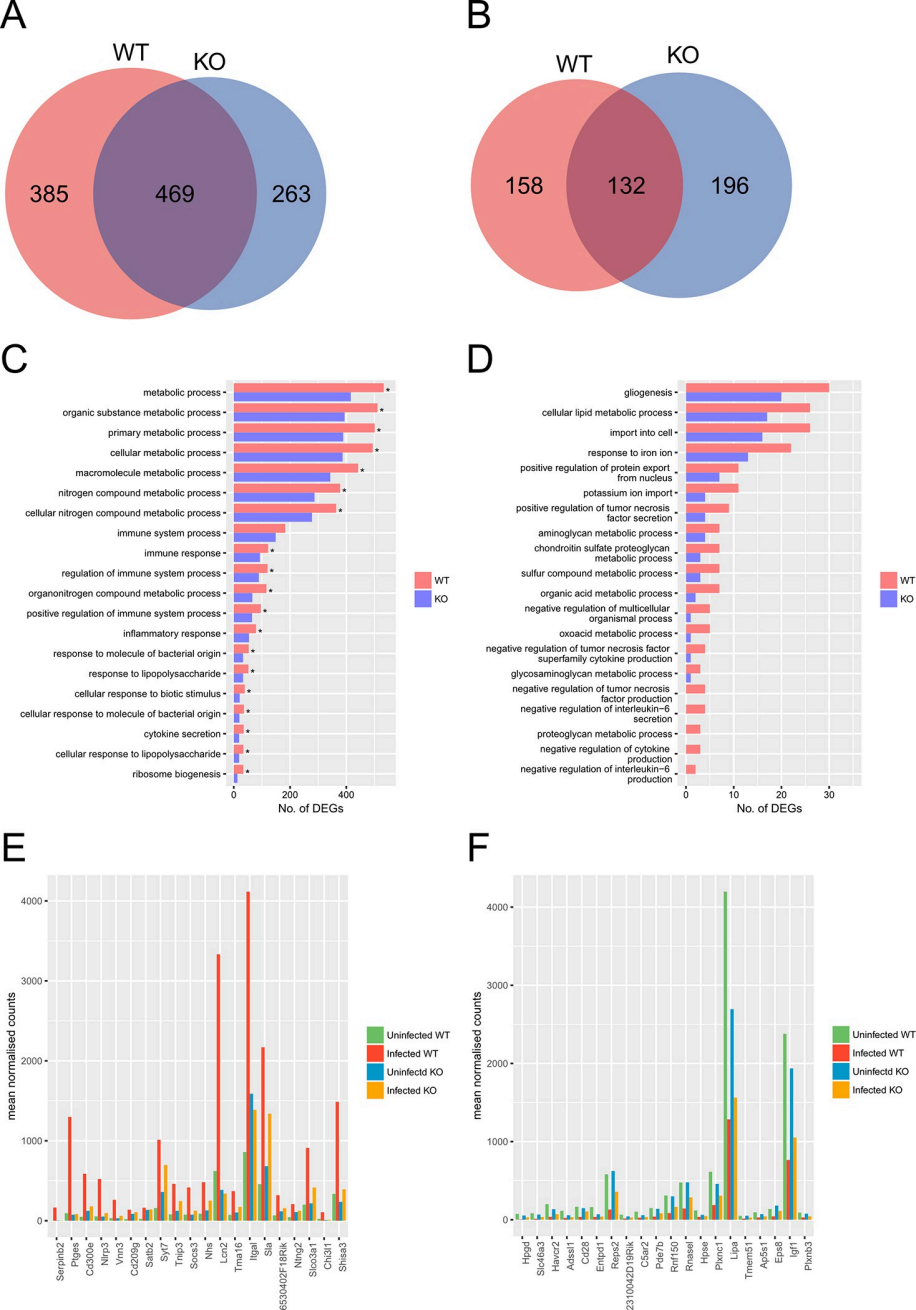
Comparison of transcriptional profiles of *Tlr2*^-/-^ and wild-type neurons during *T*. *gondii* infection. Upregulated (A, C, E) and downregulated (B, D, F) genes were identified as genes with 2-fold change and < 0.05 FDR in DESeq analysis comparing infected and uninfected cells. (A, B) Venn diagrams comparing DEGs with increased and decreased abundance between *Tlr2*^-/-^ and wild-type neurons. (C, D) To explore the function of DEGs analyzed in the Venn diagram, GO term enrichment analysis was performed. Asterisks represent significant differences with *p* < 0.05 in Fisher’s exact test. (E, F) Expression of top 20 genes highly upregulated or downregulated in a TLR2-dependent manner. TLR2-dependent DEGs were ranked according to fold-changes between infected and uninfected wild-type. WT, wild-type; KO, *Tlr2*^-/-^.

**Fig 4:**
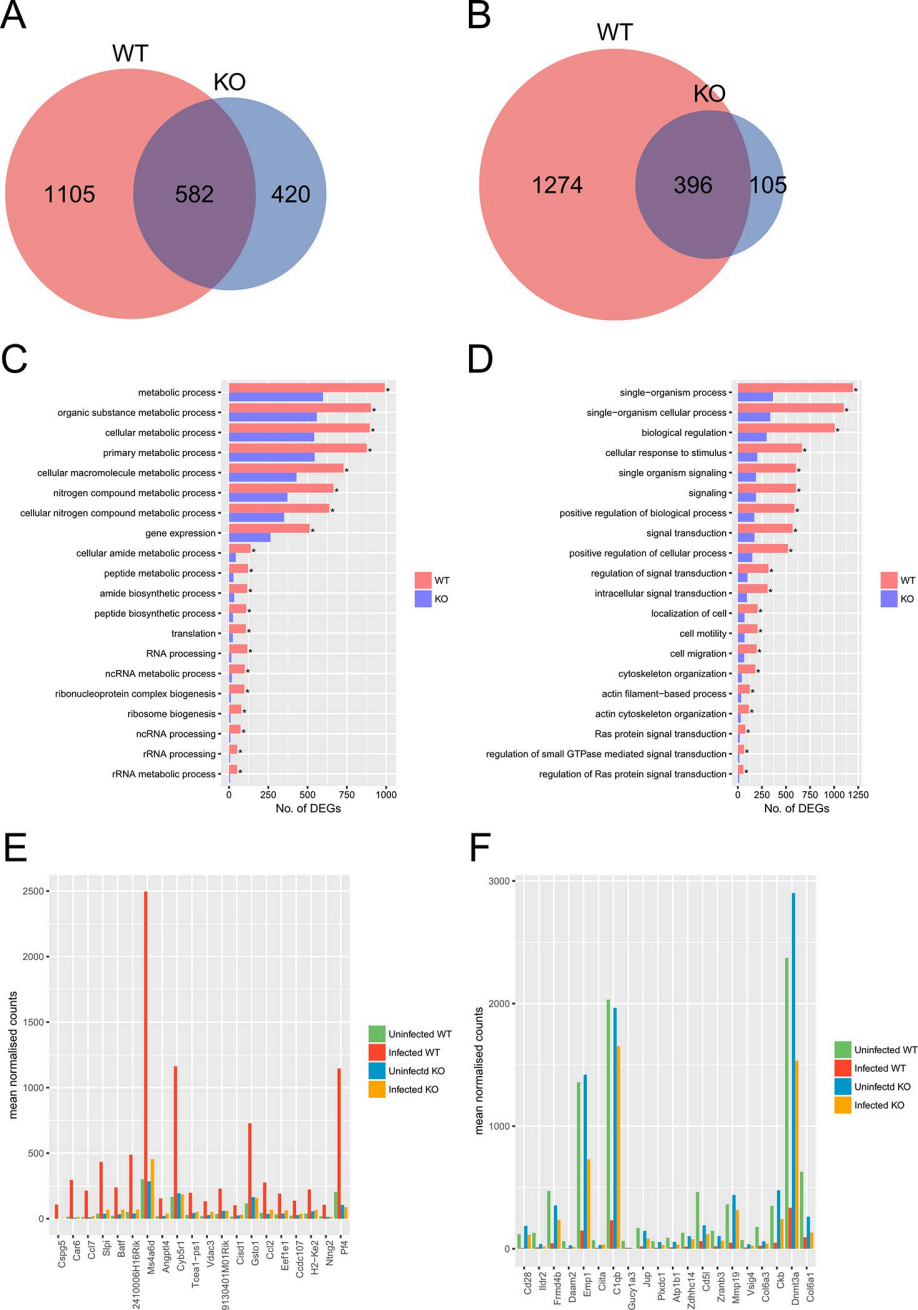
Comparison of transcriptional profiles of *Tlr2*^-/-^ and wild-type macrophages during *T*. *gondii* infection. Upregulated (A, C, E) and downregulated (B, D, F) genes were identified as genes with 2-fold change and < 0.05 FDR in DESeq analysis comparing infected and uninfected cells. (A, B) Venn diagrams comparing DEGs with increased and decreased abundance between *Tlr2*^-/-^ and wild-type macrophages. (C, D) To explore the function of DEGs analyzed in the Venn diagram, GO term enrichment analysis was performed. Asterisks represent significant differences with *p* < 0.05 in Fisher’s exact test. (E, F) Expression of top 20 highly upregulated or downregulated TLR2-depdent genes. TLR2-dependent DEGs were ranked according to fold-changes between infected and uninfected wild-type. WT, wild-type; KO, *Tlr2*^-/-^.
