# Supplementary figures and images for: Transcriptional profiling of Toll-like receptor 2-deficient primary murine brain cells during Toxoplasma gondii infection
Source: PLoS One. 2017 Nov 14;12(11):e0187703. doi: 10.1371/journal.pone.0187703 (PMC5685635; doi:10.1371/journal.pone.0187703)

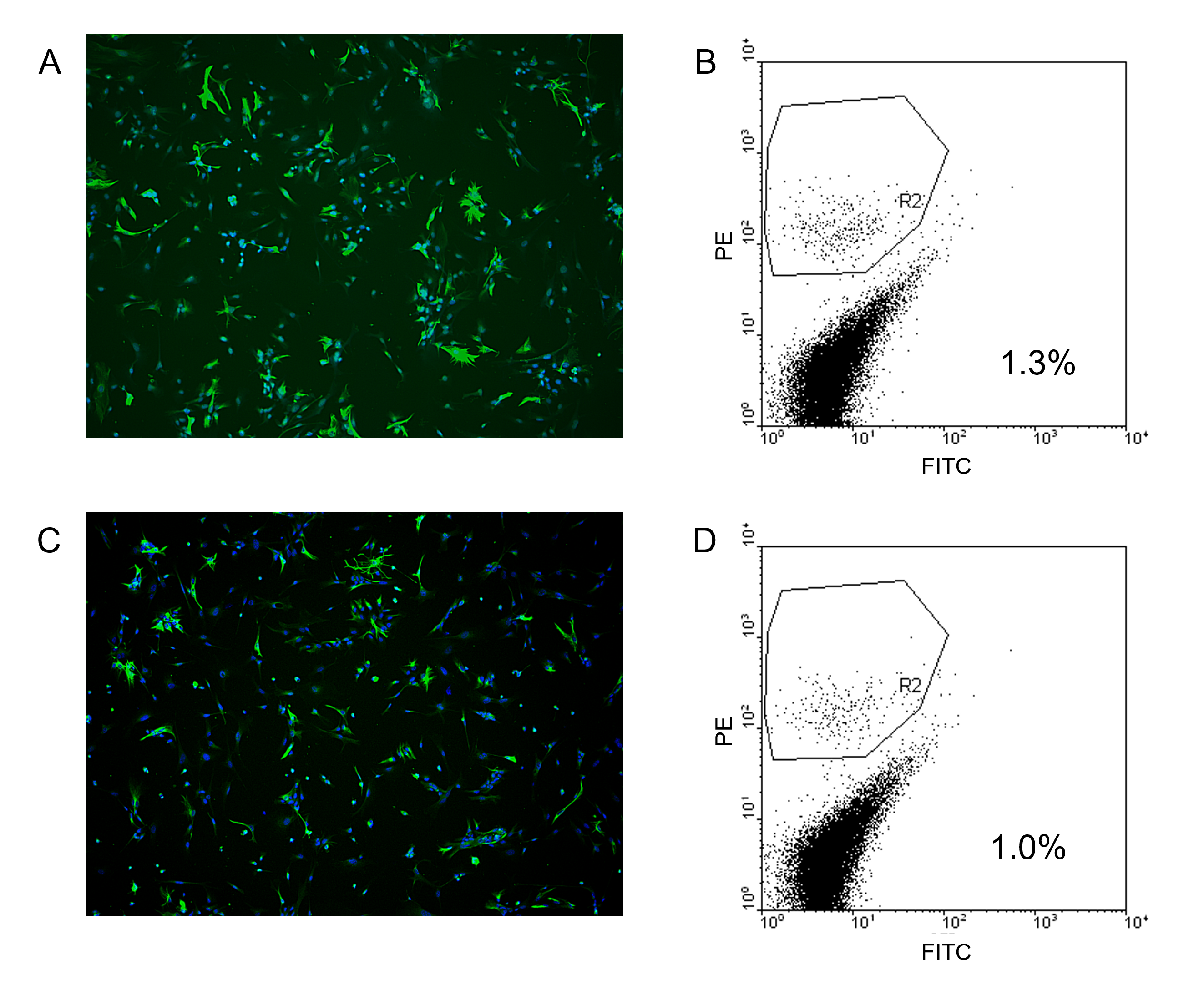

Supplement: S1 Fig — Cells positively stained for glial fibrillary acidic protein were identified as astrocytes by immunofluorescence microscopy (A, C). Flow cytometry confirmed that few of them were positively stained with phycoerythrin (PE) labeled anti-mouse CD11b antibody (y axis; B, D). A and B, wild-type; C and D, Tlr2-/-. (TIF) [file pone.0187703.s001.tif]

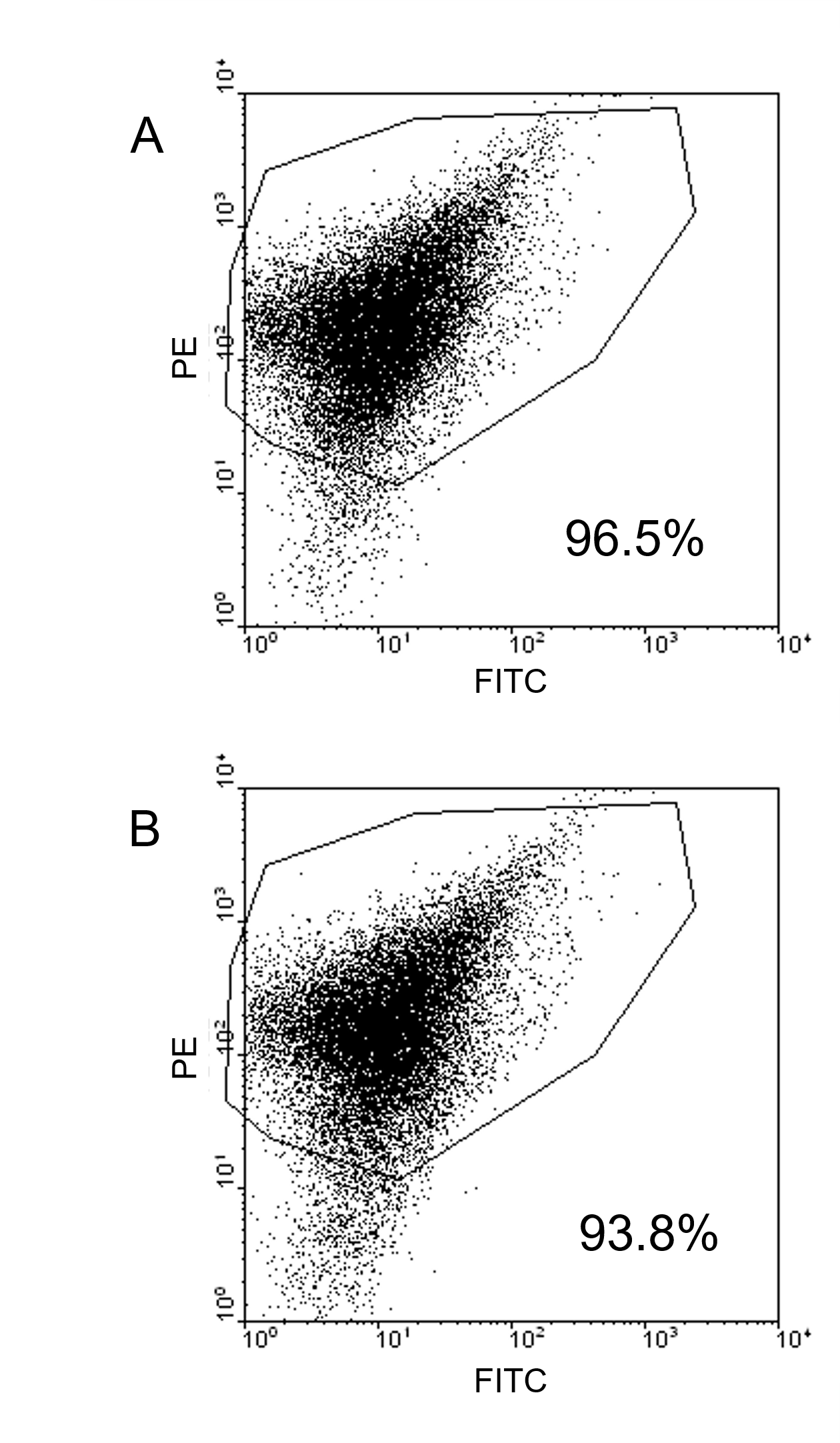

Supplement: S2 Fig — Cells positively stained with phycoerythrin (PE) labeled anti-mouse CD11b antibody (y axis) were identified as microglia by flow cytometry. A, wild-type; B, Tlr2-/-. (TIF) [file pone.0187703.s002.tif]

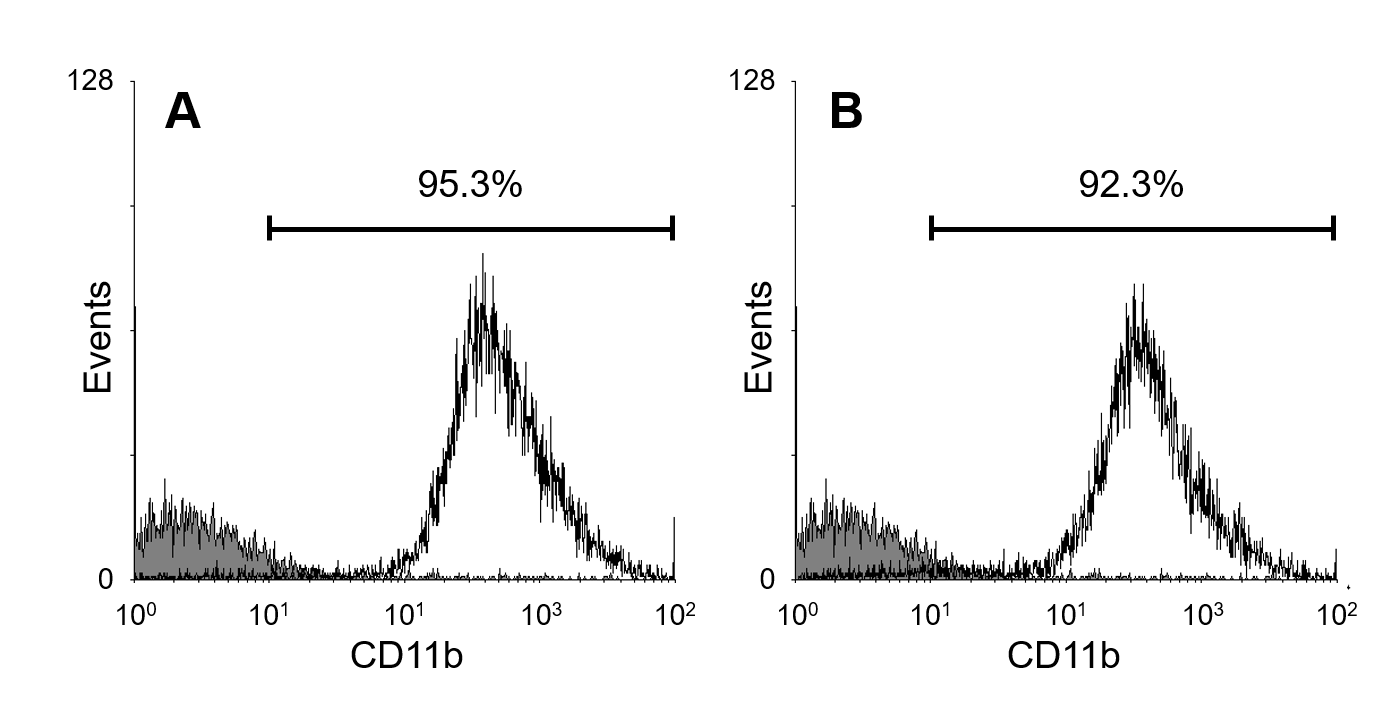

Supplement: S3 Fig — Cells positively stained with phycoerythrin (PE) labeled anti-mouse CD11b antibody (x axis) were identified as macrophages by flow cytometry. A, wild-type; B, Tlr2-/-. (TIF) [file pone.0187703.s003.tif]

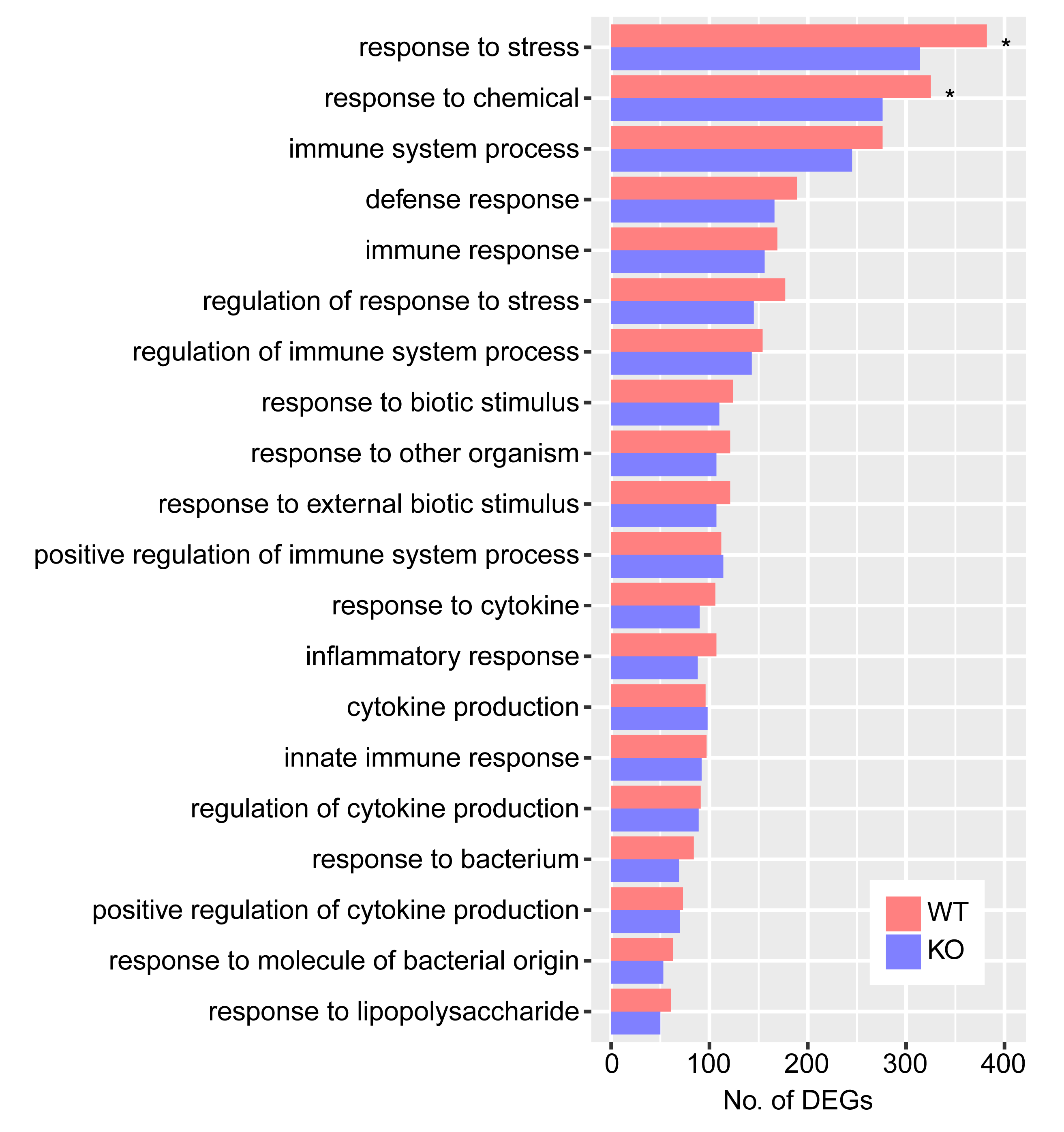

Supplement: S4 Fig — WT, wild-type; KO, Tlr2-/-. Asterisks represent significant differences with p < 0.05 in Fisher’s exact test. (TIF) [file pone.0187703.s004.tif]

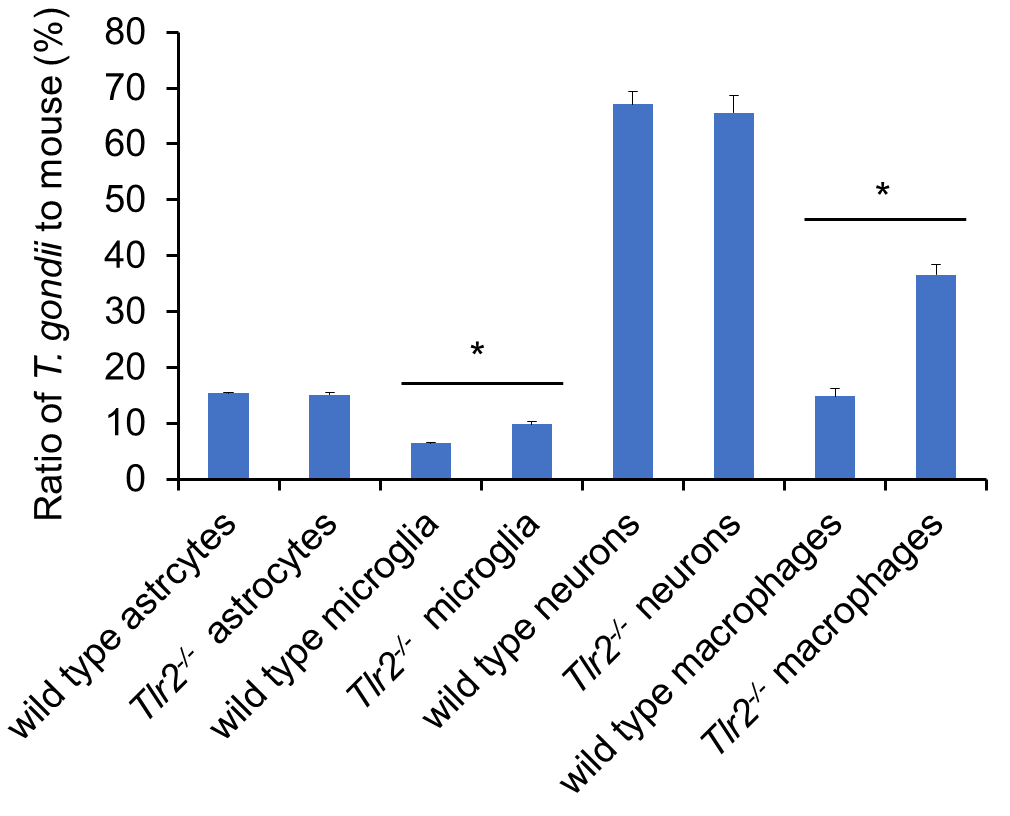

Supplement: S5 Fig — Ratio of raw read counts for T. gondii transcripts to counts for mouse transcripts were compared between wild-type and Tlr2-/- in each cell type. Asterisks represent significant differences with p < 0.05 in Student’s t-test after arcsine transformation. (TIF) [file pone.0187703.s005.tif]
